# Biocomposite Films Based on Biopolysaccharide and Hydrothermal Carbonised Pine Sawdust Waste: Properties, Performance, and Degradation Behaviour in Different Environments

**DOI:** 10.3390/molecules31203771

**Published:** 2026-10-20

**Authors:** Erika Alessia Di Liberto, Maurizio Volpe, Antonio Messineo, Nadka T. Dintcheva

**Affiliations:** 1 Dipartimento di Ingegneria, Università degli Studi di Palermo, Viale delle Scienze Ed. 6, 90128 Palermo, Italy; erikaalessia.diliberto@unipa.it; 2 Dipartimento di Ingegneria e Architettura, Università degli Studi di Enna “Kore”, Cittadella Universitaria, 94100 Enna, Italy; maurizio.volpe@unikore.it (M.V.); antonio.messineo@unikore.it (A.M.)

**Keywords:** chitosan-based films, pine sawdust hydrochar, chitosan bio-composite, properties, degradation behaviour, different environments

## Abstract

Pine sawdust waste was converted by hydrothermal carbonisation into carbon-rich particles (HTC-WS) and incorporated into chitosan to produce a sustainable biocomposite film (Chit_HTC-WS). Chitosan–microcrystalline cellulose film (Chit_MCC) and neat chitosan (Chit) were also prepared for comparison. The films were characterised by structural, mechanical, thermal, surface, UV resistance, and degradation properties in freshwater, simulated seawater, and soil burial conditions. The results obtained highlight that HTC-WS provides UV blocking and reduces transparency in the visible range, similarly to MCC. Despite their different chemical natures and elemental compositions, both HTC-WS and MCC can reduce chitosan rigidity, increasing the ductility of the film, albeit less pronouncedly, compared to the neat chitosan film. Both HTC-WS and MCC can slightly improve the photo-oxidation resistance of chitosan-based films due to their ability to absorb harmful UVB rays. Neat Chit shows rapid degradation in soil burial (up to disappearance within ca. 15 days), whereas Chit_MCC and, even more so, Chit_HTC-WS, show significantly less degradation; specifically, Chit_HTC-WS does not disappear even after 96 days, exhibiting weight loss of ca. 30%, whereas Chit_MCC disappears after 96 days. Interestingly, chitosan shows different hydrolytic degradation in freshwater and simulated seawater, and as expected, the presence of salts slows down its hydrolysis. The presence of MCC and HTC-WS reduces the hydrolytic degradation of chitosan-based films, resulting in a final weight loss of approximately 30% in both environments.

## 1. Introduction

Scientific research is increasingly focused on promoting the use of renewable and sustainable materials capable of reducing civil and industrial waste by converting it into materials that can be used in advanced applications. Within the circular economy framework, particular attention is devoted not only to renewable feedstocks but also to the conversion of waste streams to develop advanced, high-value materials with multiple applications [1,2,3]. In this context, chitosan (β-1,4-d-glucosamine), the most popular cationic polysaccharide derived from chitin, has attracted considerable attention for packaging applications due to its excellent film-forming ability, antimicrobial properties, biocompatibility, biodegradability, and non-toxicity [4]. It is an excellent candidate for use as a single material in drug delivery and biomedical applications [5,6,7], in blends with other biopolymers [8,9] and/or biocomposites containing naturally occurring fillers [10,11] for packaging and other applications [12,13]. Nevertheless, its practical use as a packaging material is still limited by its relatively poor mechanical performance or inadequate water barrier properties [4].

Several strategies have therefore been proposed to overcome these limitations. Chitosan can be blended with other polysaccharides (such as pectin, starch, cellulose, alginate, carrageenan, etc.), polyhydroxyalkanoates (such as PHA, PHB, etc.), polyethylene glycol (PEG), etc., to modify its flexibility and properties at break, making it useful for packaging applications [14,15,16,17,18]. Other strategy involves the incorporation of naturally derived reinforcing fibres and cellulose-based filler, such as cotton, flax, kenaf, cellulose nanofibers, or microcrystalline cellulose (MCC) [10,19,20,21]. The naturally derived fibres and fillers can be used without treatment or can be subjected to physical–chemical modifications to improve the adhesion between the host matrix and the additives and to enhance the final properties and performance.

Among these reinforcing phases, cellulose-based fillers are particularly attractive because of their renewability, biodegradability, relatively high stiffness, and chemical affinity with chitosan. Owing to the structural similarity between cellulose and chitosan and the presence of numerous hydroxyl and amino groups, strong intermolecular interactions can develop through hydrogen bonding, leading to improved adhesion between filler and matrix and enhanced mechanical properties [22,23,24,25,26]. In particular, microcrystalline cellulose has been reported to enhance the mechanical performance, UV-barrier properties, and surface hydrophobicity of chitosan-based films [27]. Cellulose nanocrystals and cellulose nanofibres have also been widely employed as reinforcing agents, often providing increases in tensile strength and barrier performance because of their high aspect ratio and specific surface area [28,29]. However, cellulose-based fillers also present some limitations. Their hydrophilic nature can negatively affect moisture resistance, while strong filler–filler interactions may promote aggregation, particularly at increasing filler contents, reducing effective stress transfer and potentially compromising film ductility. Moreover, highly purified or nanoscale cellulose fillers generally require additional isolation, purification, and processing steps before incorporation into polymer matrices.

These drawbacks have stimulated increasing interest in alternative fillers derived from lignocellulosic waste. Biomass-derived fillers offer different advantages, including their renewable nature, potential low cost, scalability, the possibility of controlled and eco-friendly production, and their potential for carbon sequestration. However, untreated lignocellulosic waste often shows heterogeneous composition, poor dimensional control, and high moisture sensitivity. Consequently, physical, chemical, or thermochemical treatments are commonly employed to modify their structure and to improve their interaction with other polymers. However, these treatments present several drawbacks, including the use of toxic and corrosive solvents, high energy consumption, and the generation of waste requiring additional treatment [30].

Among the possible biomass treatments, the hydrothermal carbonisation process (HTC) is increasingly recognised as an environmentally friendly and sustainable technology for the conversion of waste materials into high-value products. This approach enables the degradation of biomass primary constituents, cellulose, hemicellulose, and lignin, to produce valuable carbon-rich materials while promoting a more sustainable approach to biomass valorization using water as a solvent [30,31]. The HTC process is carried out in water under subcritical conditions, typically at temperatures between 180 and 280 °C and corresponding autogenous water pressures between 10 and 70 bar [32,33]. During the process, water acts as a catalyst, promoting several reactions, including dehydration, decarboxylation, hydrolysis, and aromatization [34]. The process produces a carbon-rich solid fraction generally referred to as hydrochar, together with a liquid phase rich in organics, and a gaseous phase mainly composed of carbon dioxide. Compared to the respective biomass, hydrochar exhibits a higher carbon along with reduced O/C and H/C atomic ratios. Overall, hydrochar is an environmentally friendly, cost-effective material with high thermal stability [35]. The hydrochar exhibits a range of advantageous properties that make it suitable for various applications in the industry and environment, including soil amendment, energy production, and activated carbon for environmental remediation [36,37,38]. Furthermore, recent studies have demonstrated the potential of hydrochar as a sustainable filler in different biopolymer-based composite systems. Nizamuddin et al. [39,40] developed polylactic acid (PLA)-based composites incorporating hydrochar derived from rice straw, reporting improvements in the chemical, structural, thermal, mechanical, and rheological properties of the polymer matrix. Similarly, Ye et al. [41] produced composites by blending poly (butylene adipate-co-terephthalate) (PBAT) with hydrochar derived from wood chips. The HC/PBAT composites have shown enhanced thermal stability and mechanical performance.

Despite these developments, the use of hydrochar in polysaccharides systems has not been used so far as a plastic additive for packaging applications. Hydrochar from nettle has been incorporated as a filler to cellulose nanofibers (CNF) for the development of CNF-based packaging films for food preservation. Khan et al. [42] demonstrate that CNF-based composites have the potential to preserve product quality and extend shelf life thanks to the antimicrobial, antioxidant, and UV-blocking properties of hydrochar.

To the best of our knowledge, the incorporation of wood-derived hydrochar as a filler into chitosan-based films has not yet been explored, highlighting its potential as a plastic additive for packaging applications. In this context, pine sawdust waste was subjected to HTC treatment to produce hydrochar (HTC-WS) particles to be used as an additive for the production of chitosan bio-based films. The HTC-WS particles were characterised in terms of thermal stability (TGA), morphological features (SEM), particle size distribution, surface chemical functionalities using FTIR-ATR analysis, proximate analysis, and elemental and calorimetric analysis.

The aim of this work is the formulation of chitosan-based films using the solvent casting technique, incorporating hydrochar as a reinforcing and functional filler. For comparative purposes, reference films based on neat chitosan (Chit), and on chitosan and commercial microcrystalline cellulose (Chit_MCC), were formulated under similar conditions. To enhance the adhesion between cellulose particles and the biopolymer matrix, glycerol was incorporated as a compatibiliser during the formulation of the films. Moreover, this work investigates the mechanical and degradation properties of new chitosan biocomposite films formulated with the addition of hydrochar particles rich in cellulose obtained from pine sawdust waste in comparison to the commercial microcrystalline cellulose. The morphological, mechanical, and durability (e.g., resistance to UV rays) of the chitosan-based films were analysed, assessing possible changes in structure, transparency, and performance over time. Furthermore, the biodegradation ability of the chitosan-based films in different environments was evaluated. The samples were subjected to hydrolytic degradation in freshwater and simulated seawater, and the soil burial test and biodegradability were monitored by weight loss.

The novelty of this work is the development of a new biocomposite based on chitosan and hydrochar derived from pine sawdust waste as an alternative to the commercial microcrystalline cellulose. This strategy represents a promising approach for the development of materials with potential functional advantages but also addresses growing environmental concerns by promoting the valorisation of lignocellulosic waste. In this context, the use of natural by-products contributes to reducing dependence on non-renewable resources, minimising waste disposal, and supporting the transition from a conventional linear economy toward a more sustainable circular model.

## 2. Results and Discussion

### 2.1. Characterisation of Hydrothermal Carbonised Sawdust Particles (HTC-WS)

**Figure 1:**
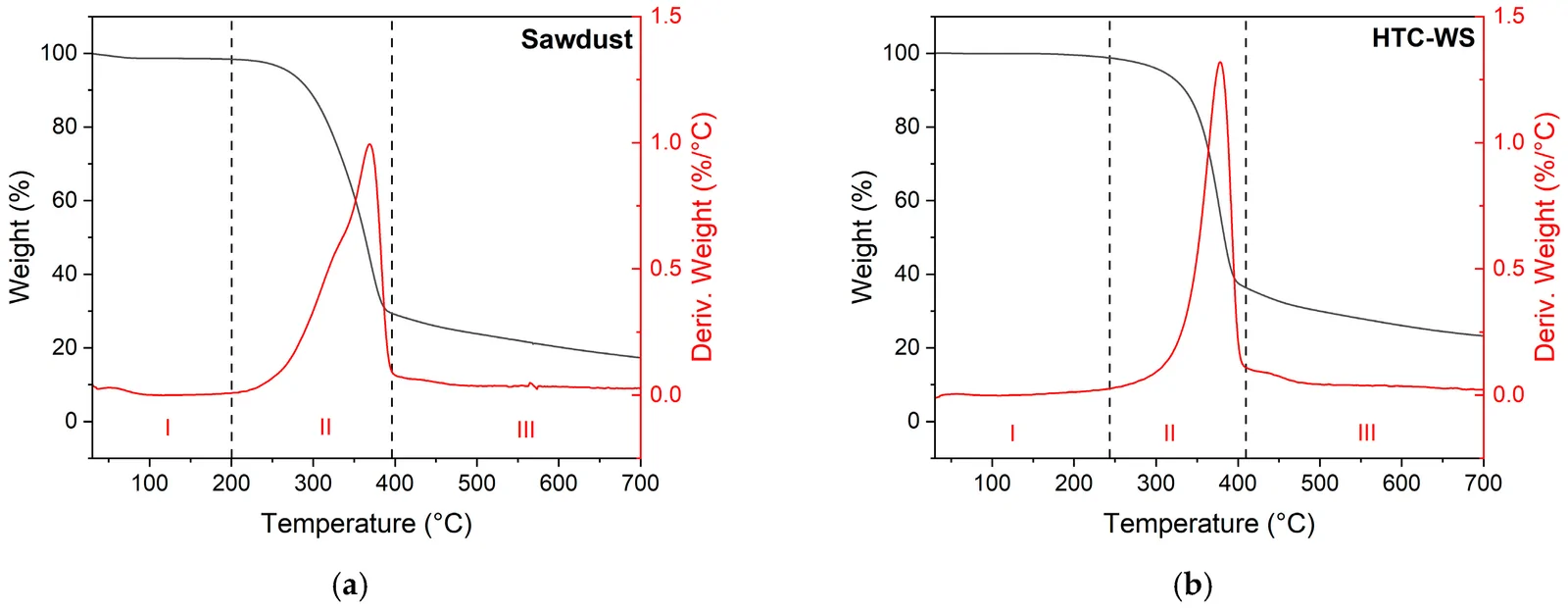
TGA/DTG thermograms of sawdust (**a**) before and (**b**) after the hydrothermal carbonisation (HTC) process.

The thermal stability of the pine sawdust and the HTC-WS particles obtained after the HTC treatment were analysed using thermogravimetric analysis. Figure 1a,b shows the results of the analysis both before and after the HTC treatment. It is worth noting that in the HTC process, a temperature of 210 °C was specifically chosen to preserve the cellulose content of the sawdust, according to the literature [43,44]. Indeed, Volpe et al. [34] investigated the HTC of pure cellulose and birchwood to analyse the reactivity of cellulose within lignocellulosic biomass. Their results demonstrated that cellulose remained essentially unaltered at temperatures up to 220 °C, whereas significant decomposition occurred at 230 °C.

The thermograms in Figure 1a,b reveal, also according to the literature [30], three distinct stages and thermal transitions; specifically, the first stage occurs from 30 °C to around 200 °C, the second stage from around 200 to 400 °C, and the final stage from 400 °C to around 700 °C.

In Figure 1a, a weight loss of around 2% was observed in the first stage, corresponding to the removal of residual moisture. The second stage, with a weight loss of about 55%, extends up to around 400 °C due to the decomposition of hemicellulose and cellulose. Although the thermal decomposition of both hemicellulose and cellulose overlaps within the same signal, the largest mass loss is attributed to the decomposition of cellulosic compounds. The final stage, starting around 400 °C, is associated with the decomposition of lignin and results in a residue of approximately 17% at 700 °C.

In Figure 1b, no weight loss is observed in the first region up to 200 °C, indicating that no moisture is present and the HTC-WS particles are thermally stable up to approximately 220 °C. Moreover, the second stage of HTC-WS occurs at higher temperatures than that of raw sawdust. Specifically, the onset of the second stage is observed at around 200 °C for sawdust and around 250 °C for HTC-WS. In this stage, a single peak is detected, corresponding to cellulose decomposition, with a weight loss of approximately 43%. In the final stage, a residue of approximately 23% is observed at 700 °C, probably due to the formation of a thermally recalcitrant, high-energy-density aromatic material. The results shown in Figure 1b indicate a reduction and partial removal of hemicellulose, accompanied by an increase in the relative cellulose content after HTC treatment.

Furthermore, different values of the maximum weight loss rate temperature (T_max_) were observed for the sawdust and HTC-WS particles. Specifically, the T_max_ and corresponding weight loss (%) are around 370 °C and 56% for pine sawdust, and around 378 °C and 43% for HTC-WS. The results show that the HTC-WS particles obtained are more thermally stable than the untreated raw material.

According to the literature [45], although cellulose and lignin are not removed during the HTC process at ca. 210 °C, it is worth noting that carbon-rich particles may be successfully used to improve mechanical properties when used to reinforce the bioplastics.

**Figure 2:**
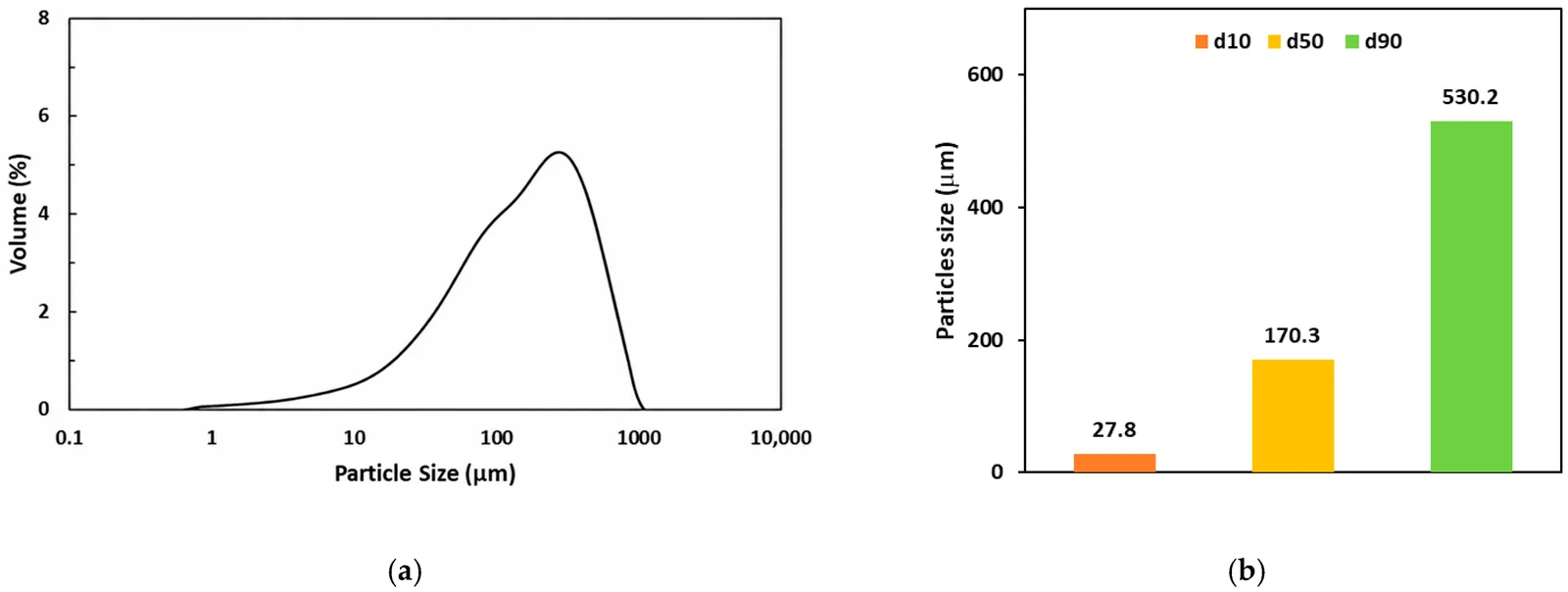
(**a**) Size distribution and (**b**) fractions of HTC-WS particles produced.

The dimensional distribution curve of the HTC-WS particles obtained was analyzed using a particle-size analyser, as shown in Figure 2a. The d10, d50, and d90 factors were also calculated, representing the maximum diameter values of 10%, 50%, and 90% of the particles, respectively. The particle-size distribution indicates that 10% of the HTC-WS particles have a diameter of approximately 28 μm (d10), 50% have a diameter of approximately 170 μm (d50), and 90% have a diameter of approximately 530 μm (d90). In contrast, sawdust exhibits particle sizes ranging from tens of micrometres to a few millimetres.

The mass yields, energy properties, and proximate analysis results of HTC-WS are summarised in Table 1. Mass yields, HHV, energy yields, ash, and fixed carbon (FC) contents changed after the hydrothermal carbonisation reaction. During the hydrothermal treatment of lignocellulosic biomass, hemicellulose exhibits higher reactivity than cellulose, whereas lignin is comparatively recalcitrant to degradation [46]. The proximate analysis results show a MY_HC_ of 71.8%, MY_L_ of 25.8%, and 2.4% of MY_G_. In addition, the FC content increased from 19.43% to 25.46%, while the volatile matter (VM) content of sawdust decreased from 80.32% to 74.44%. A reduction in ash content was also observed after hydrothermal carbonisation, which can be attributed to the removal of a significant fraction of inorganic species present in the raw pine sawdust during the HTC process [47]. The HHV is one of the most important parameters for characterizing fuel properties, which was calculated from the elemental composition and ash content of the sawdust and corresponding hydrochar. As a result of higher carbon content and lower VM, the HHV of HTC-WS was higher than that of the corresponding raw biomass. Overall, the hydrothermal carbonisation process led to an increase in HHV, thereby enhancing the fuel properties of the materials.

Elemental analysis results indicate that the hydrothermal carbonisation process did not significantly influence the hydrogen content, whereas a slight increase in nitrogen content and a more pronounced increase in carbon content were observed in HTC-WS in comparison to pine sawdust. Specifically, the nitrogen content increased from 0.16% to 0.91%, while the carbon content increased from 46.3% to 51.4%, for sawdust and HTC-WS, respectively. The increments are attributed mainly to the removal of oxygenated volatile components during the HTC process, leading to a relative enrichment of carbon and nitrogen in the HTC-WS particles.

**Figure 3:**
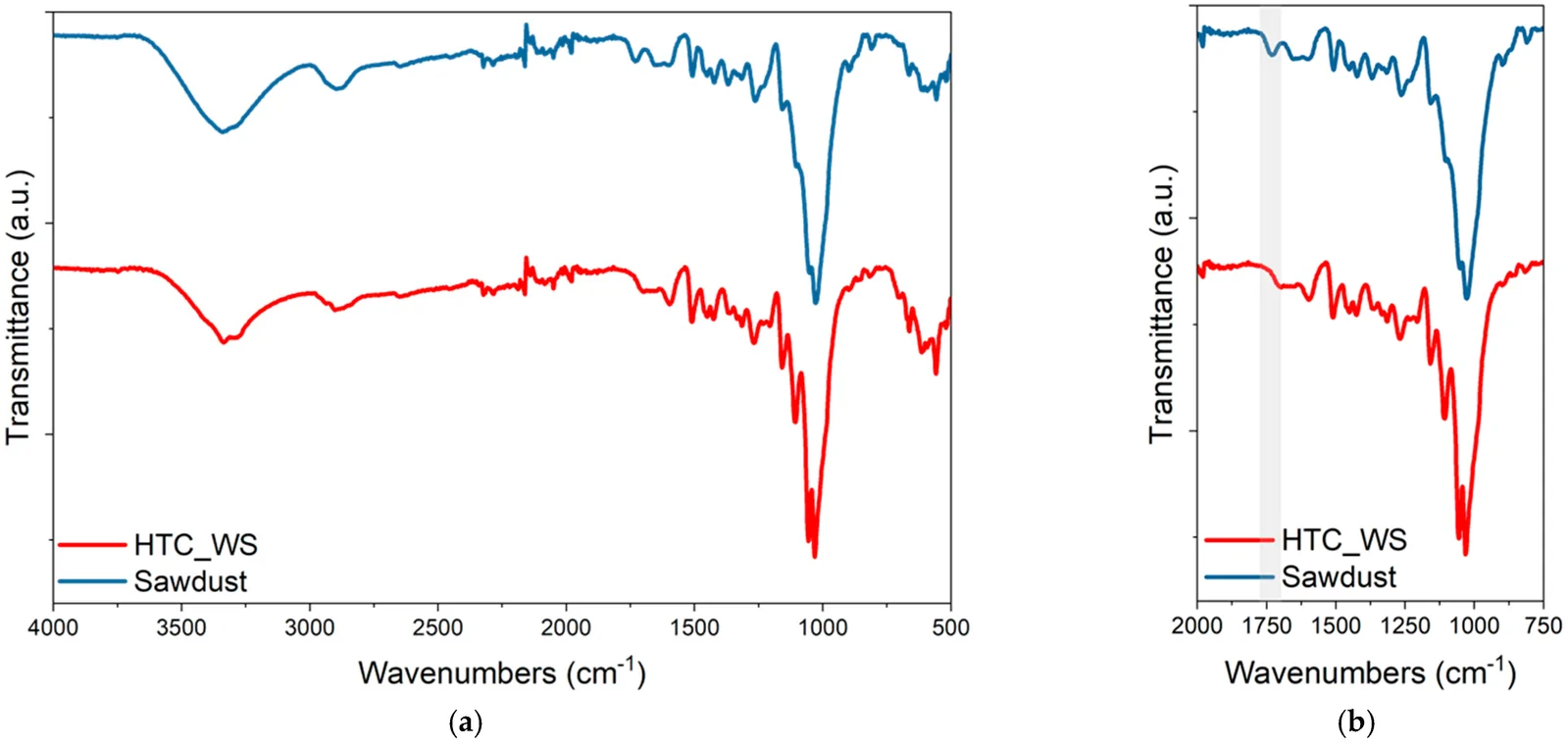
ATR-FTIR spectra of sawdust (blue line) and HTC-WS particles (red line): (**a**) whole spectra 4000–500 cm^−1^ and (**b**) selected range 2000–750 cm^−1^.

To identify the functional groups, present in the pine sawdust and HTC-WS and to assess the possible structural changes induced by the HTC treatment, ATR-FTIR spectroscopy analysis was performed. The spectra shown in Figure 3a exhibit a broad peak between 3600 and 3000 cm^−1^, which is associated with the stretching vibration of aliphatic O-H groups, including hydroxyl and carboxyl groups. The peak observed in the 1100–1000 cm^−1^ range corresponds to the C-O stretching vibration of phenols, esters, and aliphatic alcohols, while the peak at approximately 1200 cm^−1^ is associated with O-H bending vibrations. The reduced intensity of the hydroxyl and carboxyl peaks in the HTC-WS particles indicates that dehydration and decarboxylation reactions occurred during the HTC process [45,48].

Cellulose degradation was not detected due to its thermal stability up to approximately 230 °C [43,44,49]. The characteristic C=O stretching band of hemicellulose, observed at around 1729 cm^−1^ in the spectra of raw pine sawdust, disappeared in the spectra of HTC-WS; see Figure 3b. This disappearance provides clear evidence of hemicellulose degradation occurring above 200 °C, under hydrothermal conditions [50]. The peaks in the 1650–1500 cm^−1^ range correspond to the C=O and C=C stretching vibrations of the aromatic rings in lignin. Although with reduced intensity, these bands persisted after the carbonization process, indicating the presence of lignin fragments and intermediate structures in the HTC-WS particles, implying that lignin did not undergo complete degradation under the investigated conditions [51].

**Figure 4:**
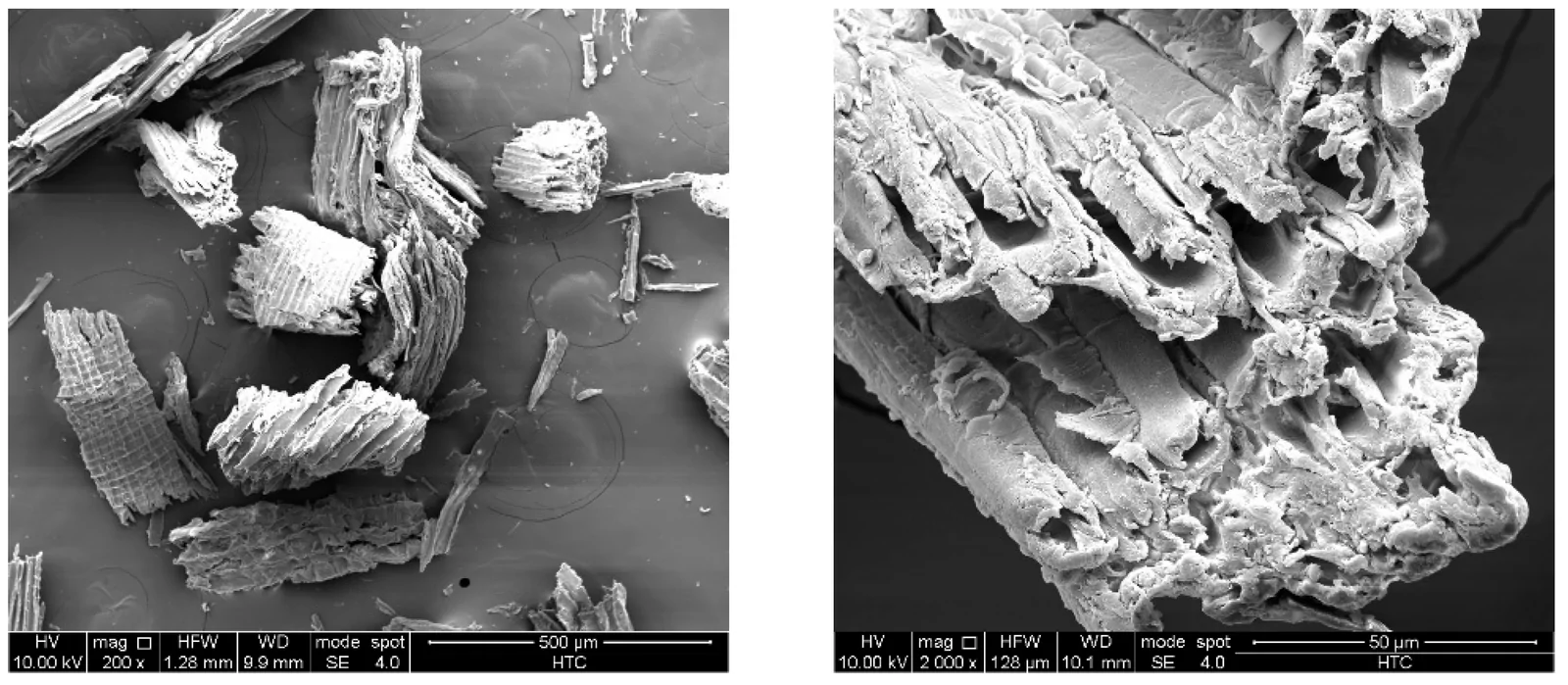
SEM micrographs of HTC-WS particles at different magnifications.

Morphological analysis was carried out to examine the morphology of the HTC-WS obtained. The micrographs in Figure 4 of the HTC-WS particles at different magnifications show a typical lignocellulosic morphology, characterised by jagged surfaces, pores, and preserved wood cell structures.

Indeed, a closer examination of the particle morphology revealed that these types of materials are mainly composed of bundles of tubules forming a circular cross-section, exhibiting the characteristic hierarchical architecture typical of lignocellulosic biomass.

The observed surface irregularities may be associated with the removal of hemicellulose content during the HTC process. These irregularities could potentially favor interactions with polymer matrices when the particles are used as fillers in biocomposite formulations.

### 2.2. Characterisation of Biocomposite Chitosan-Based Films

Chitosan-based films were successfully produced by the solvent casting technique and subjected to spectroscopy (UV-visible and ATR-FTIR), water contact angle (WCA), calorimetry (DSC), tensile test (elastic modulus, tensile strength, and elongation at break), and microscopy (SEM) analyses.

**Figure 5:**
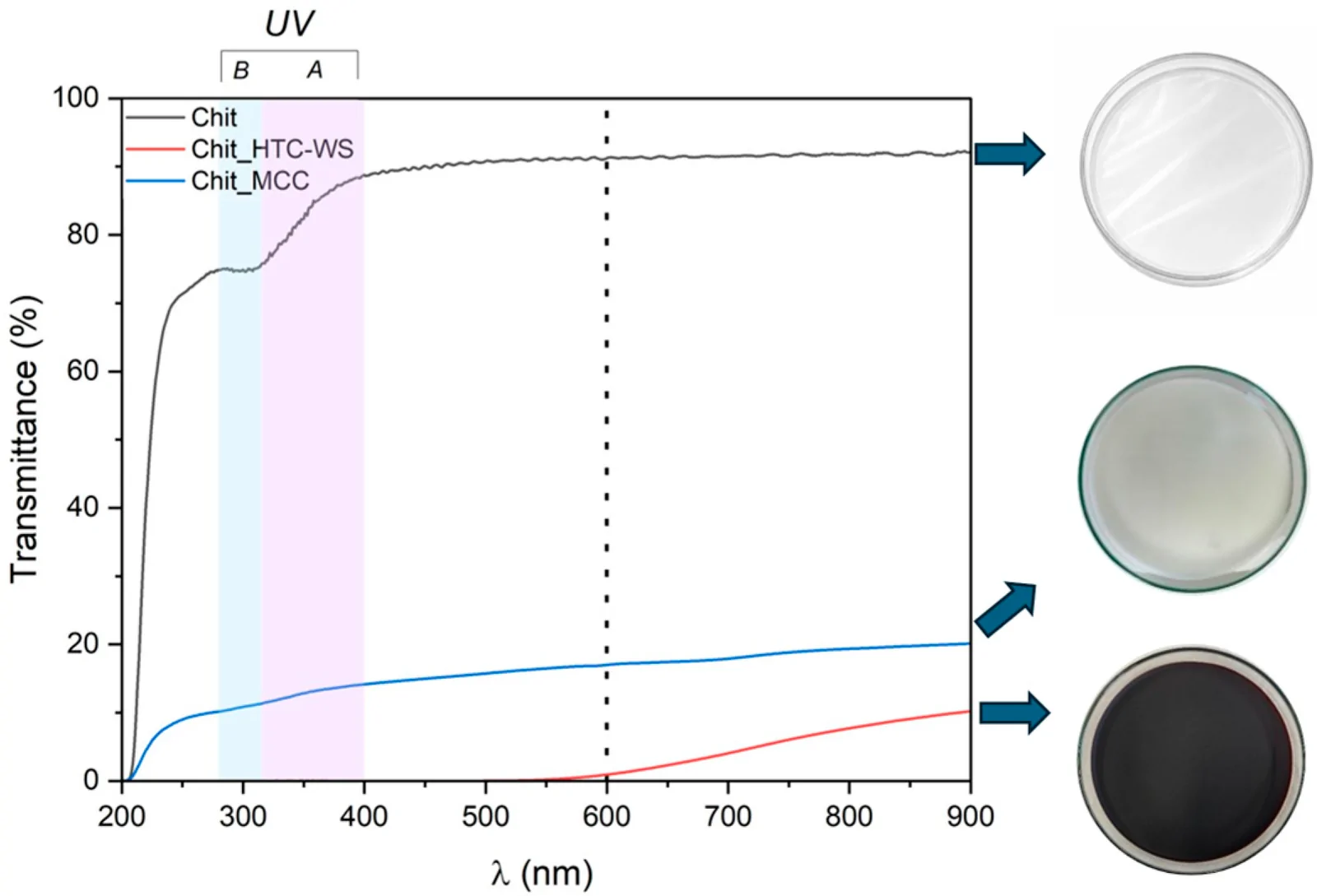
UV-vis spectra of Chit film (black line), Chit_HTC-WS film (red line), and Chit_MCC (blue line) and overview of the films (images).

The UV-blocking performance of the chitosan-based composite films was evaluated using UV–Vis spectroscopy to assess the effect of incorporating HTC-WS particles into the polymer matrix, also in comparison to microcrystalline cellulose (MCC). The obtained UV-visible spectra of neat chitosan and chitosan-based biocomposite films, containing HTC-WS and MCC, are presented in Figure 5. The blue and pink shaded areas highlight the UV-B (280–315 nm) and UV-A (315–400 nm) spectral regions, respectively, allowing a clearer visualization and comparison of the UV-shielding behavior of the different films. According to the literature [52,53], the UV–vis spectrum of the chitosan film exhibits a broad peak in the 270–350 nm region, which can be attributed to oxidation processes occurring during film formation by solvent casting.

Transparency (T) values were calculated according to Equation (6), as reported in the experimental section, based on the measured absorbance values at 600 nm and the respective film thicknesses (mm). All the values obtained are reported in Table 2, which enables a direct comparison of the optical properties of the neat Chit, Chit_HTC-WS, and Chit_MCC biocomposite films. This overview highlights how the incorporation of different fillers influences parameters such as transparency, light transmittance, and overall visual appearance.

According to Equation (6), a higher transparency (T) value indicates a lower degree of transparency. The chitosan film was found to be more transparent with a calculated T value of 0.4 A mm^−1^. However, adding HTC-WS and MCC affected the transparency of the films, as a considerable lowering in T600 nm was registered. Indeed, the chitosan-based biocomposite films were found to be less transparent due to the presence of the natural colouring pigment present in the filler (brown HTC-WS and white MCC particles).

Despite being less transparent, the filler provides protection against UV rays. Indeed, the Chit film exhibits UV blocking activities of 16.6% and 25.1% for UV-A and UV-B rays, respectively. The Chit_MCC film exhibits UV blocking activities of 87.1% and 89.2%, respectively, which are significantly higher than the values of the neat Chit film. Interestingly, Chit_HTC-WS film demonstrates total UV blocking activity, suggesting that the addition of HTC-WS particles provides total protection against UV rays.

UV rays play a significant role in the oxidation process of various chemical reactions, thereby accelerating the degradation of many materials. Consequently, UV blocking activity is considered a desirable characteristic of packaging materials, as it helps to preserve product quality and extend shelf life by limiting irradiation exposure. In this context, the developed chitosan-based composite films can be expected to provide enhanced, or even complete, protection against UV-A and UV-B rays. These results highlight the potential of the formulated biocomposites as promising candidates for sustainable packaging applications. It is worth noting that HTC-WS particles are much more efficient than MCC in blocking UV rays.

Water contact angle (WCA) measurements were performed to assess the effect of HTC-WS and MCC particles on film wettability. The results obtained are summarised in the last column of Table 2. The WCA values recorded were approximately 80.5° for the chitosan film, 88.2° for Chit_HTC-WS, and 92.0° for Chit_MCC. As is noticeable, WCA increased in the presence of HTC-WS, indicating the formation of a more hydrophobic surface. Specifically, the HTC-WS influences the hydrophobic properties of the resulting film by reducing the tendency to absorb water molecules.

**Figure 6:**
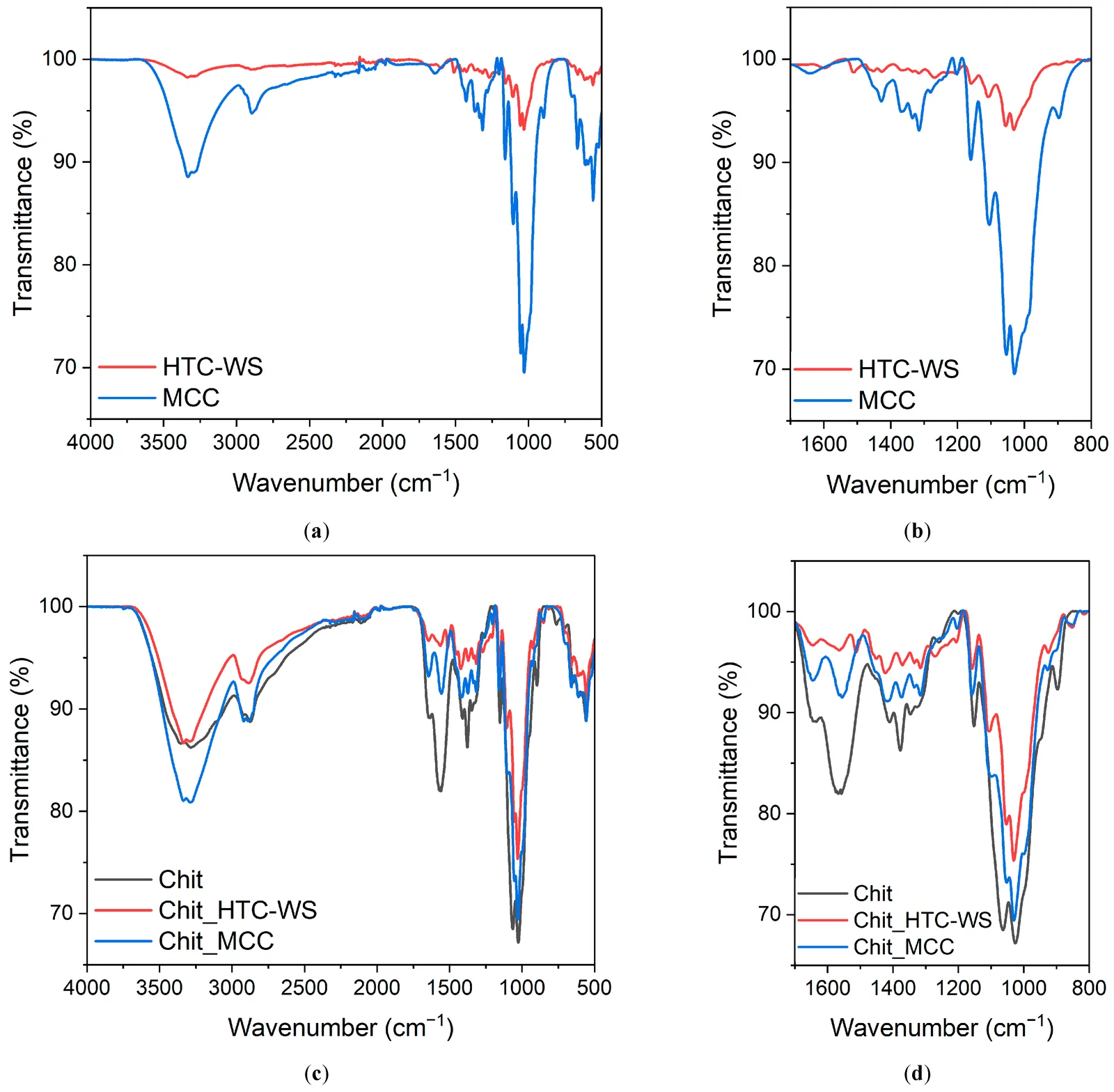
ATR-FTIR spectra of (**a**–**b**) HTC-WS and MCC powders, and (**c**–**d**) chitosan-based film: Chit (black line), Chit_HTC-WS (red line), and Chit_MCC (blue line).

The main functional groups present in the HTC-WS and MCC in powder form, before solvent-casting, were identified through FTIR analysis performed in ATR mode; see Figure 6a,b. In addition, to examine the structural interactions and potential modifications occurring within the chitosan-based composites, ATR-FTIR analyses on Chit, Chit_HTC-WS, and Chit-MCC films were conducted, and the resulting spectra are shown in Figure 6c,d.

As shown in Figure 6a, the FTIR spectra of HTC-WS and MCC exhibit similar characteristic absorption bands, which can be attributed to the comparable chemical structures. According to the literature, the characteristic bands of chitosan include the OH and NH stretching bands at 3435 cm^−1^ and between 3300–3150 cm^−1^, respectively; the C=O stretching band at 1655 cm^−1^; the -NH_2_ scissoring and NH deformation bands at 1590 cm^−1^ and 1560 cm^−1^, and the COC-stretching band, which is attributed to the glycosidic bond or pyranose ring at 1150 cm^−1^ [54]. The broad band located in the 3600–3100 cm^−1^ region is associated with O-H stretching vibration, while the band at 2895 cm^−1^ corresponds to C–H stretching [55]. The peak observed at 2872 cm^−1^ is attributed to the C–H stretching vibrations typical of polysaccharides. In addition, the other characteristic peaks of chitosan were observed at 1417 cm^−1^ (–CH_2_ bending), 1375 cm^−1^ (-CH_3_ symmetrical deformation), 1150 cm^−1^ (antisymmetric C–O–C stretching and C–N stretching), and 1026 cm^−1^ (skeletal vibrations of C–O stretching) [4]. The peak in the 1650 and 1500 cm^−1^ region is attributed to C=C stretching vibrations of aromatic rings in lignin. The spectrum of MCC shows distinctive peaks characteristic of native cellulose I. The peak observed at approximately 1641 cm^−1^ is related to the bending vibration of the H-O-H bonds of the absorbed water molecules in cellulose, with an intensity dependent on the residual hemicellulose content in MCC [56,57,58], and also to the C=O stretching of the secondary amide group in chitosan [52]. The peaks at 1428 and 897 cm^−1^ are assigned to the asymmetric –CH_2_ wagging and C–H rocking vibrations in cellulose, respectively. The presence of glycerol is evidenced in the 1400–1200 cm^−1^ and 1125–1100 cm^−1^ regions, attributed to the O-H and CH-OH stretching vibration, respectively. The broad peak observed in the 3700–2800 cm^−1^ region is associated with combined stretching vibrations of O-H and N-H groups in the biopolymer and indicates hydrogen bonding interactions between chitosan and cellulosic fillers. In both composite films, this complex band shifted toward higher wavenumbers compared with neat chitosan film, suggesting a modification of the hydrogen-bonding environment and the establishment of intermolecular interactions between the fillers and the chitosan matrix. The occurrence of intermolecular interactions indicate good compatibility between the fillers and chitosan, which may contribute to the enhanced performance of the composite materials [59,60]. The absence of new distinct absorption bands suggests that incorporation of HTC-WS does not involve the formation of new covalent bonds, but rather predominantly intermolecular interactions, particularly hydrogen bonding, between the filler and the chitosan matrix.

**Figure 7:**
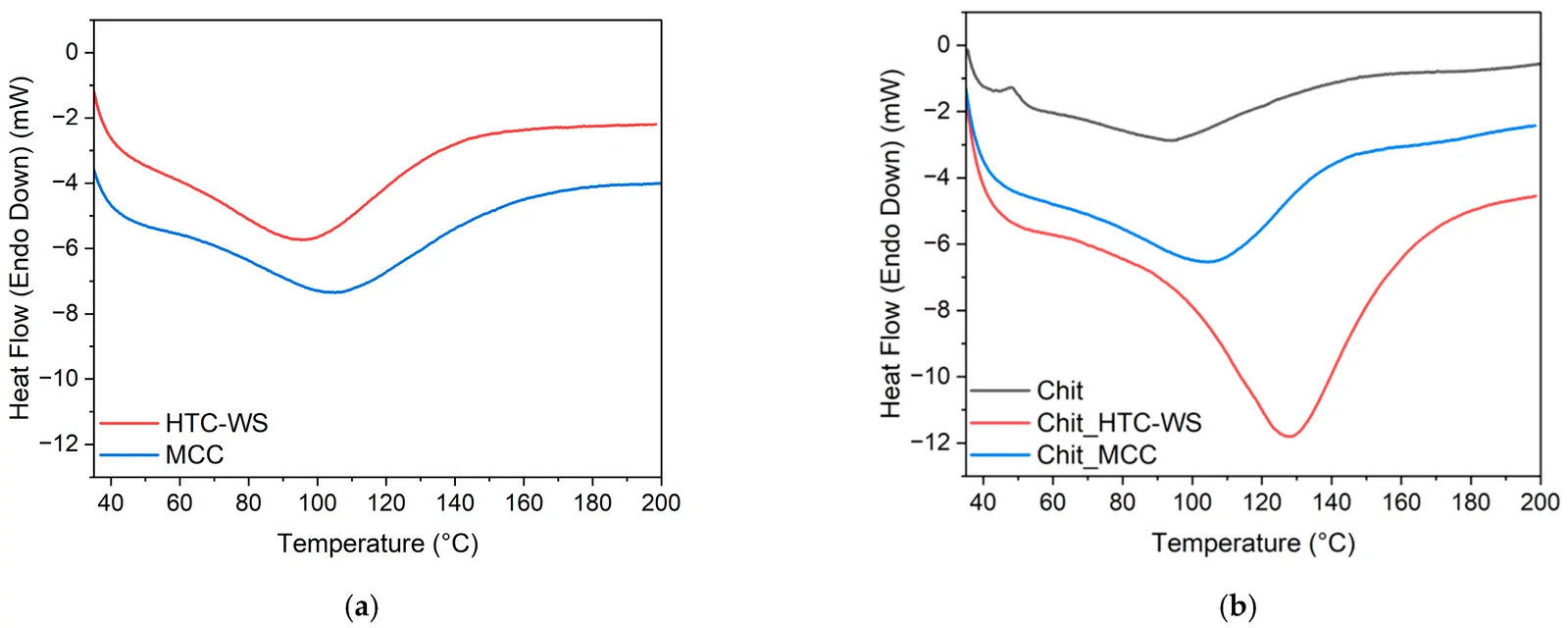
Differential scanning calorimetry (DSC) thermograms recorded during the first heating scan of (**a**) HTC-WS (red line) and MCC (blue line) powders, (**b**) chitosan-based biocomposite films: Chit film (black line), Chit_HTC-WS (red line), and Chit_MCC (blue line). (The data on Chit and Chit_MCC films were published before [53]).

To analyze the thermal behavior of HTC-WS and MCC powders and composite materials, DSC analysis, with two heating cycles and one cooling cycle, was carried out. The results indicate that only the first heating scan provides relevant information regarding the thermal history of the materials. Figure 7a,b shows the thermograms of the first heating scan of HTC-WS and MCC (Figure 7a) and chitosan-based film (Figure 7b), while Table 3 summarizes the main thermal properties (i.e., onset melting temperature (T_on_), melting temperature (T_m_), offset melting temperature (T_off_), and fusion enthalpy (ΔH)).

During the first heating scan, for all investigated materials, one thermal event was observed in the 40–200 °C range. HTC-WS and MCC powders exhibit a single thermal event associated with the water molecules coordinated by cellulose rings, occurring at 95.2 °C and 105.2 °C, for HTC-WS and MCC, respectively. The corresponding enthalpy values of 189.88 J g^−1^ and 205.14 J g^−1^, for HTC-WS and MCC, suggest that endothermic phenomena require similar energy values.

The Chit_HTC-WS and Chit_MCC biocomposite films exhibit enhanced thermal stability compared to the neat chitosan films, a result attributed to the combined influence of the incorporated fillers and the plasticizer, and this is more pronounced for the film produced with HTC-WS particles. As illustrated in Figure 7b, the incorporation of HTC-WS hydrochar particles into the chitosan film delays the solvent evaporation event, shifting the corresponding peak to higher temperatures and increasing the associated fusion enthalpy values compared to the neat chitosan film. Specifically, the average event temperature increased to 128.2 °C compared to the 94.1 °C peak observed for the neat Chit film.

**Figure 8:**
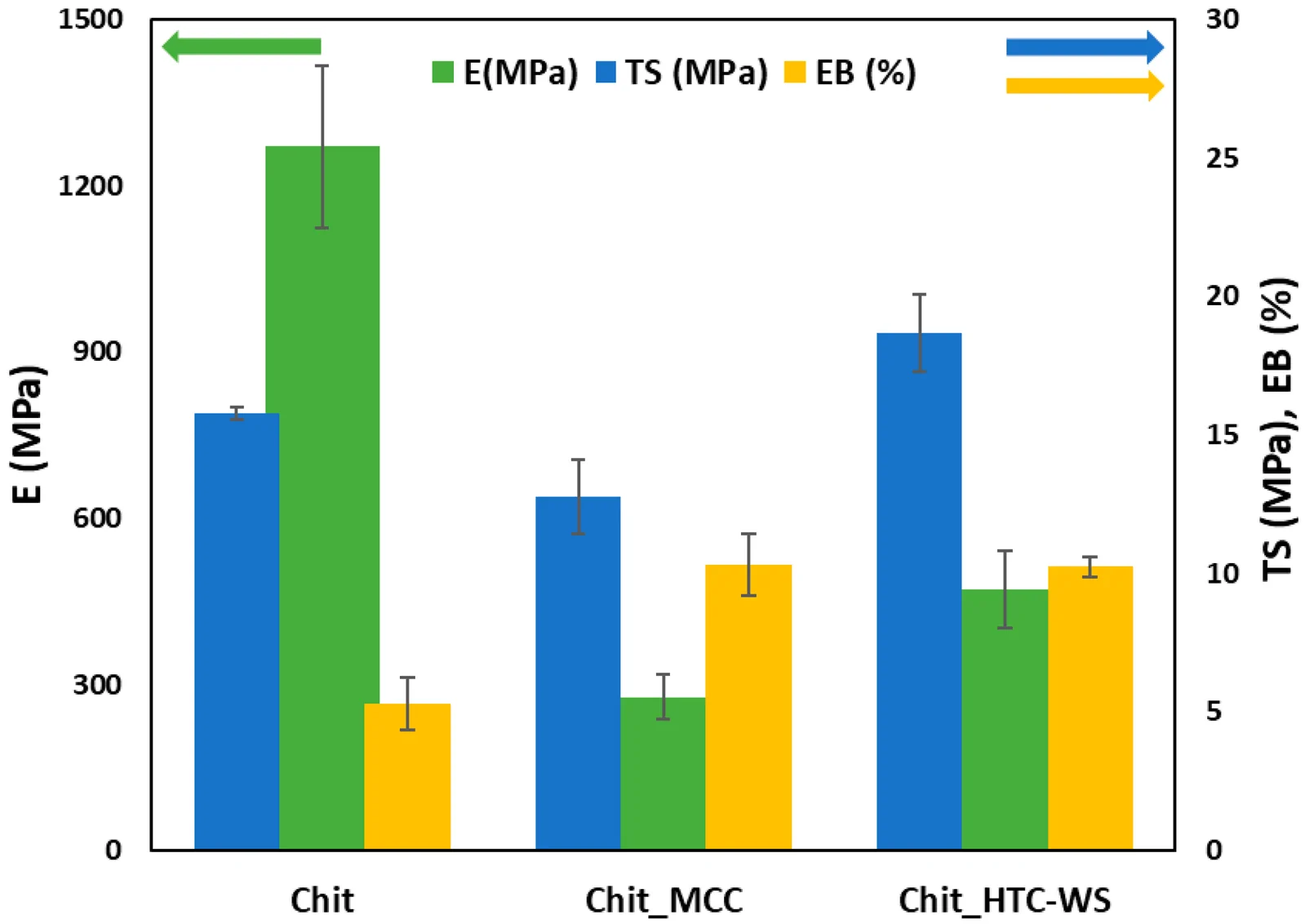
Average values of main mechanical properties of investigated biocomposite films: Elastic Modulus (MPa) (green), Tensile Strength (MPa) (blue), and Elongation at Break (%) (yellow).

The mechanical performance of the biocomposite films was evaluated by performing tensile tests on all the samples. This analysis allowed for the determination of the main mechanical properties, including the elastic modulus (E), tensile strength (TS), and elongation at break (EB), which provide a comprehensive understanding of the films’ stiffness, strength, and ductility, respectively. The results obtained are summarized in Figure 8, offering a comparative overview of the mechanical behaviour of the different biocomposite materials.

Neat chitosan films are generally characterised by their stiffness, low flexibility, and brittle nature, which limit their use in packaging applications. To overcome these drawbacks, glycerol was incorporated into the biocomposite formulations as a plasticising agent to enhance the interaction between the polymer matrix and the filler particles. Indeed, glycerol molecules can penetrate biopolymer chains and interfere with their intermolecular interactions. According to the literature [4], glycerol reduces intermolecular interactions and enhances polymer chain mobility, thereby leading to increased flexibility and ductility of the material. This effect is reflected in the measured elongation at break (EB) values, which increased from 5.3% for the neat Chit film to around 10% for the Chit_HTC-WS and Chit_MCC biocomposite films. Queirós et al. [61] mention that the formation of hydrogen bonds between the polymer chains and the additive further promotes structural cohesion and prevents phase separation, contributing to the overall mechanical integrity and improved flexibility of the biocomposite films.

Due to the abundant hydrophilic functional groups present on its surface, chitosan can form intermolecular hydrogen bonds with cellulose, resulting in the formation of stable composite materials. Furthermore, the mechanical properties of the films were modified by the HTC-WS and MCC particles, and significant differences between the formulations were observed. Specifically, differences in Young’s modulus (E) and tensile strength (TS) were observed between the chitosan film and the chitosan-based films. Adding HTC-WS particles to the film increased its tensile strength compared to the control Chit film from 15.8 MPa to 18.7 MPa. It is worth mentioning that, although the Chit_MCC film exhibited elongation at break (EB) values comparable to those of the Chit_HTC-WS sample, its E and TS values were considerably lower, indicating that the reinforcing effect of MCC was less pronounced than that provided by HTC-WS particles.

**Figure 9:**
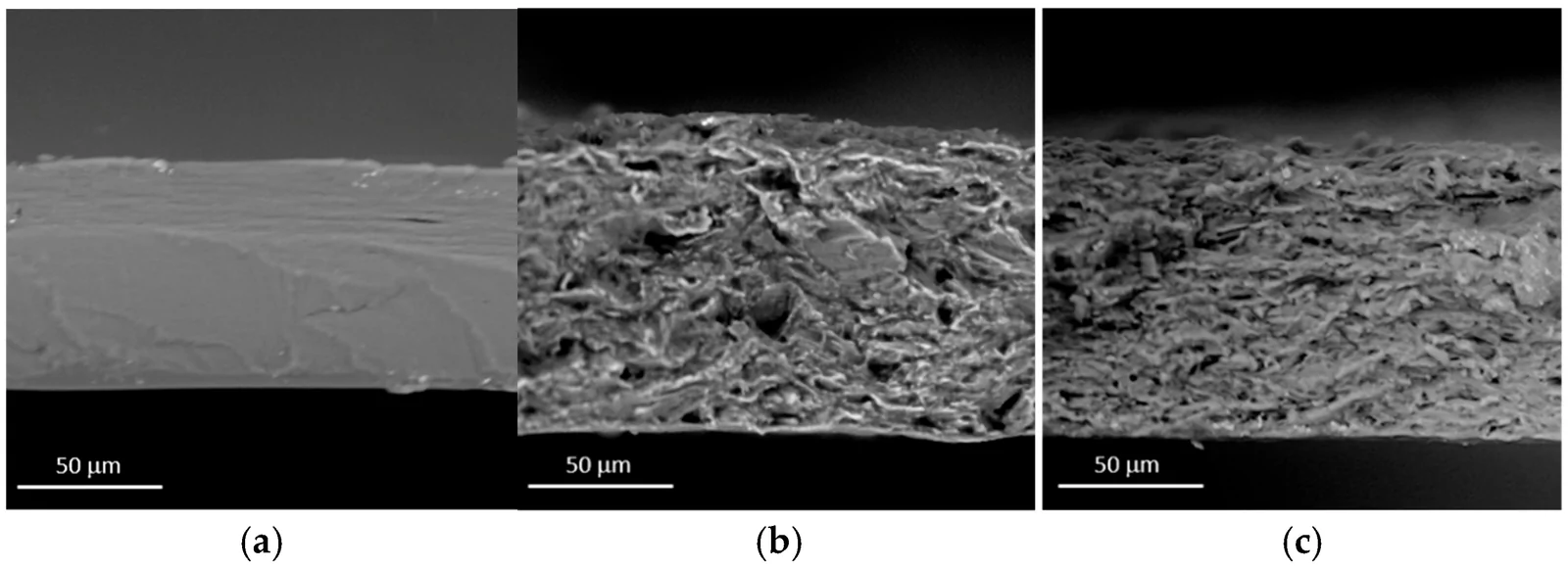
Micrographs of (**a**) Chit film, (**b**) Chit_HTC-WS film, (**c**) Chit_MCC film.

Morphological analysis was performed to evaluate the filler dispersion and the interfacial adhesion of the biocomposite materials. The cross-sectional micrographs of the Chit, Chit_HTC-WS, and Chit_MCC films are presented in Figure 9a–c.

As observed in Figure 9b,c, the surfaces of the chitosan-based composite films are rougher in comparison to the neat chitosan film. This increased roughness is attributed to the incorporation of fillers into the chitosan matrix, which enhances resistance to external stresses. Consequently, higher tensile strength values are observed for the composite films, as confirmed by the mechanical analysis. Indeed, the surfaces of both Chit_HTC-WS and Chit_MCC biocomposite films appear very similar, suggesting that HTC-WS is a suitable and effective filler for the preparation of chitosan-based composite films.

### 2.3. Photo-Oxidation Resistance and Hydrolytic Degradation in Different Environments

**Figure 10:**
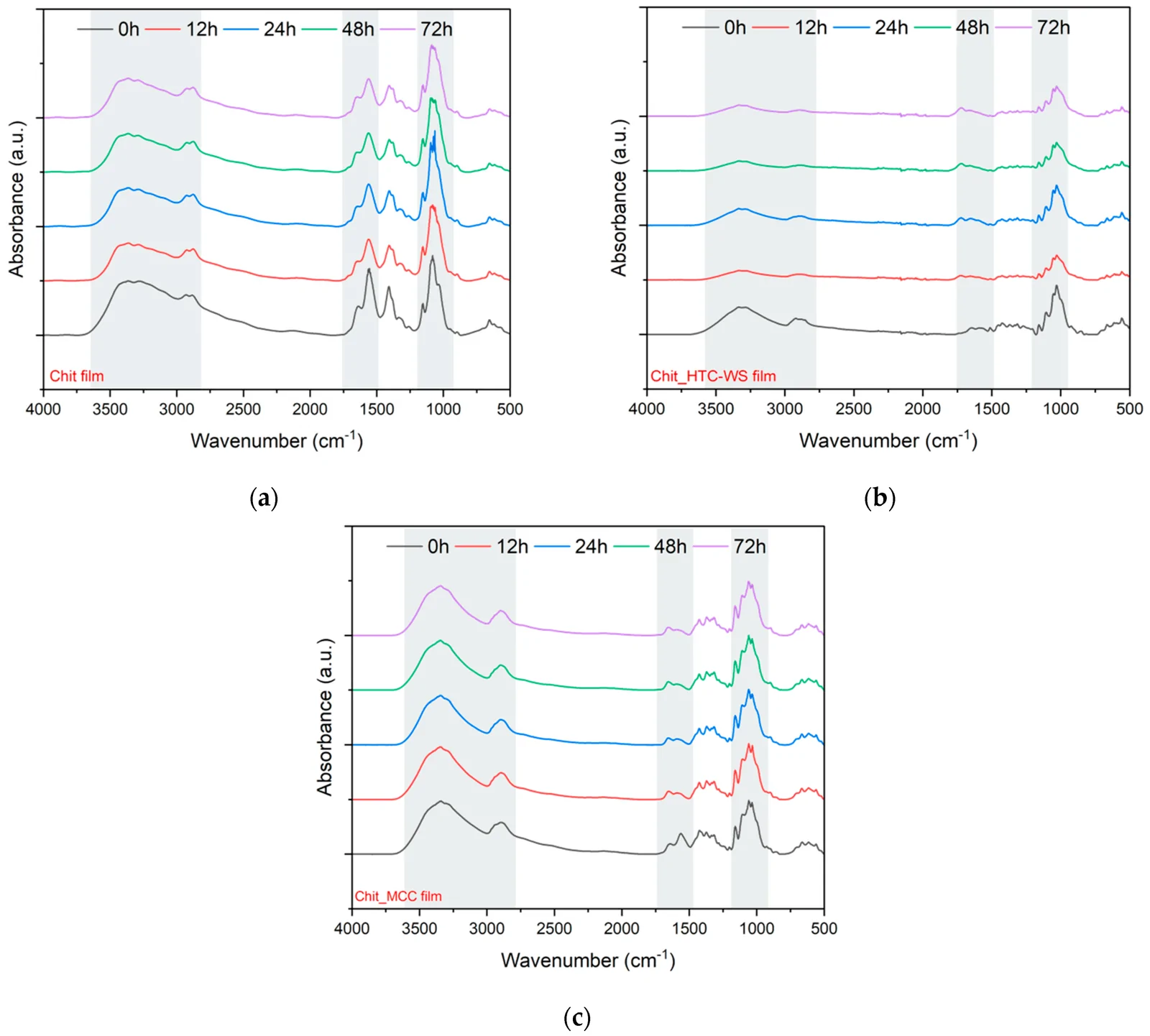
FTIR-ATR spectra of (**a**) Chit film, (**b**) Chit_HTC-WS, and (**c**) Chit_MCC biocomposite films at different exposure times.

Neat chitosan films and chitosan-based biocomposite films were subjected to accelerated photo-oxidation to simulate degradation phenomena under service conditions. The progression of photo-oxidative degradation over time was monitored by FTIR analysis, and the spectra collected at different exposure times are presented in Figure 10a–c.

According to literature [52,54,62], the degradation mechanism of chitosan primarily involves depolymerization, followed by deacetylation and, in the presence of oxygen, oxidation, as well as random interchain crosslinking.

Irradiation of the chitosan film (Figure 10a) provoked a marked decrease in the absorption in the domain 3500–3300 cm^−1^ due to the loss of constitutive water or to the breaking of hydrogen bonds between the macromolecules.

Although the FTIR spectra of chitosan-based films before photo-oxidation (0 h) are very similar, a slight decrease in absorbance in the 2000–1500 cm^−1^ region was observed for both systems after 24 h of exposure. This decrease can likely be attributed to the loss of coordinated water molecules during the initial stage of irradiation. Moreover, the FTIR spectra of Chit_MCC (Figure 10c) exhibit a reduction in absorbance at 1150 cm^−1^ over time, which reflects the disappearance of the pyranose ring/acetal bond and can be correlated with the concurrent formation of oxidation products, evidenced by the increment of the band at 1730 cm^−1^ [54]. This behavior suggests a higher resistance to photo-oxidation of Chit_HTC-WS film compared to that of Chit_MCC. Overall, these results indicate that the incorporation of HTC-WS particles provides a beneficial protective effect on chitosan-based biocomposite films against photo-oxidative degradation under UVB exposure.

**Figure 11:**
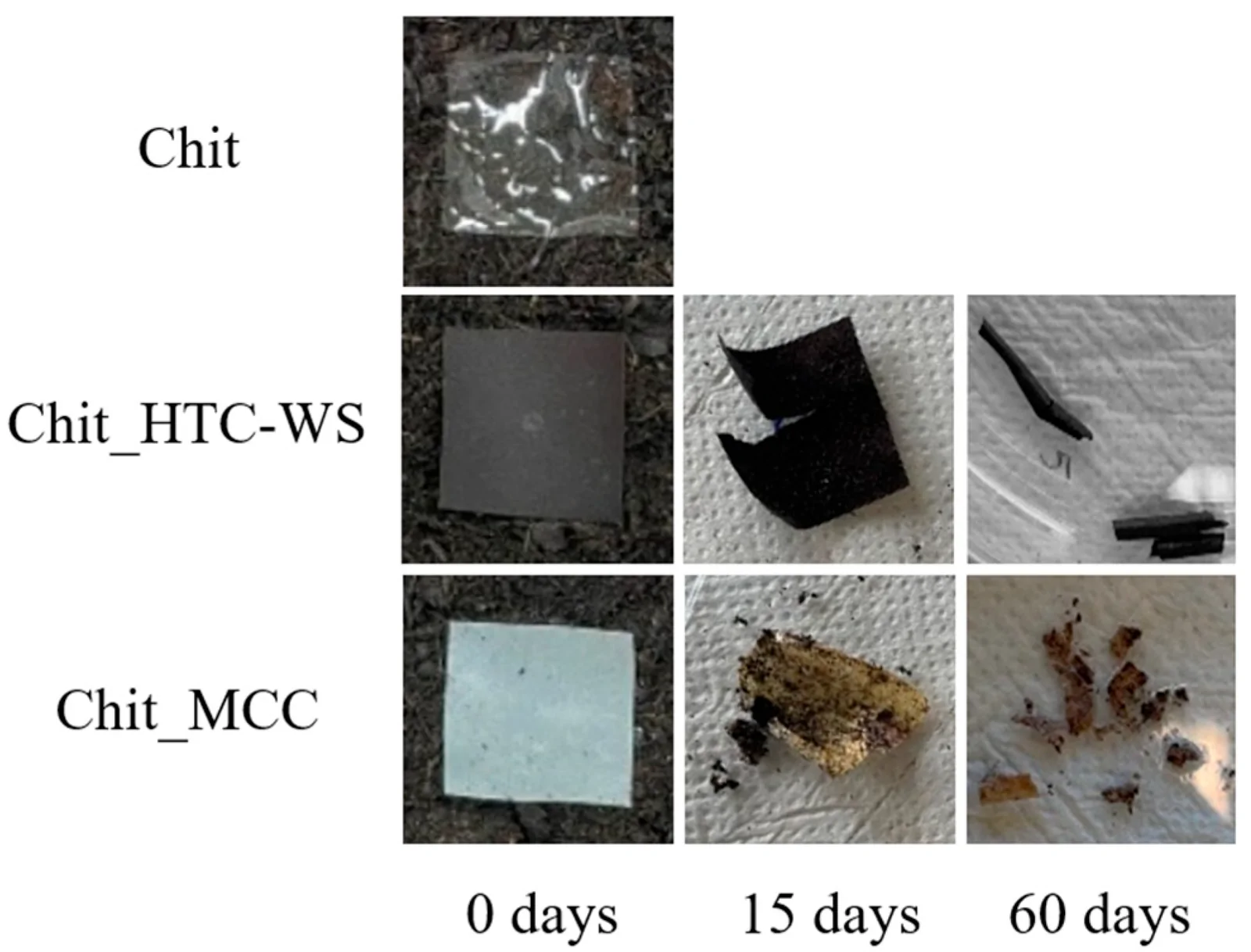
Images of the visual appearance of the chitosan and chitosan-based films: before incubation, after 15 days, and after 60 days of incubation in the soil.

**Figure 12:**
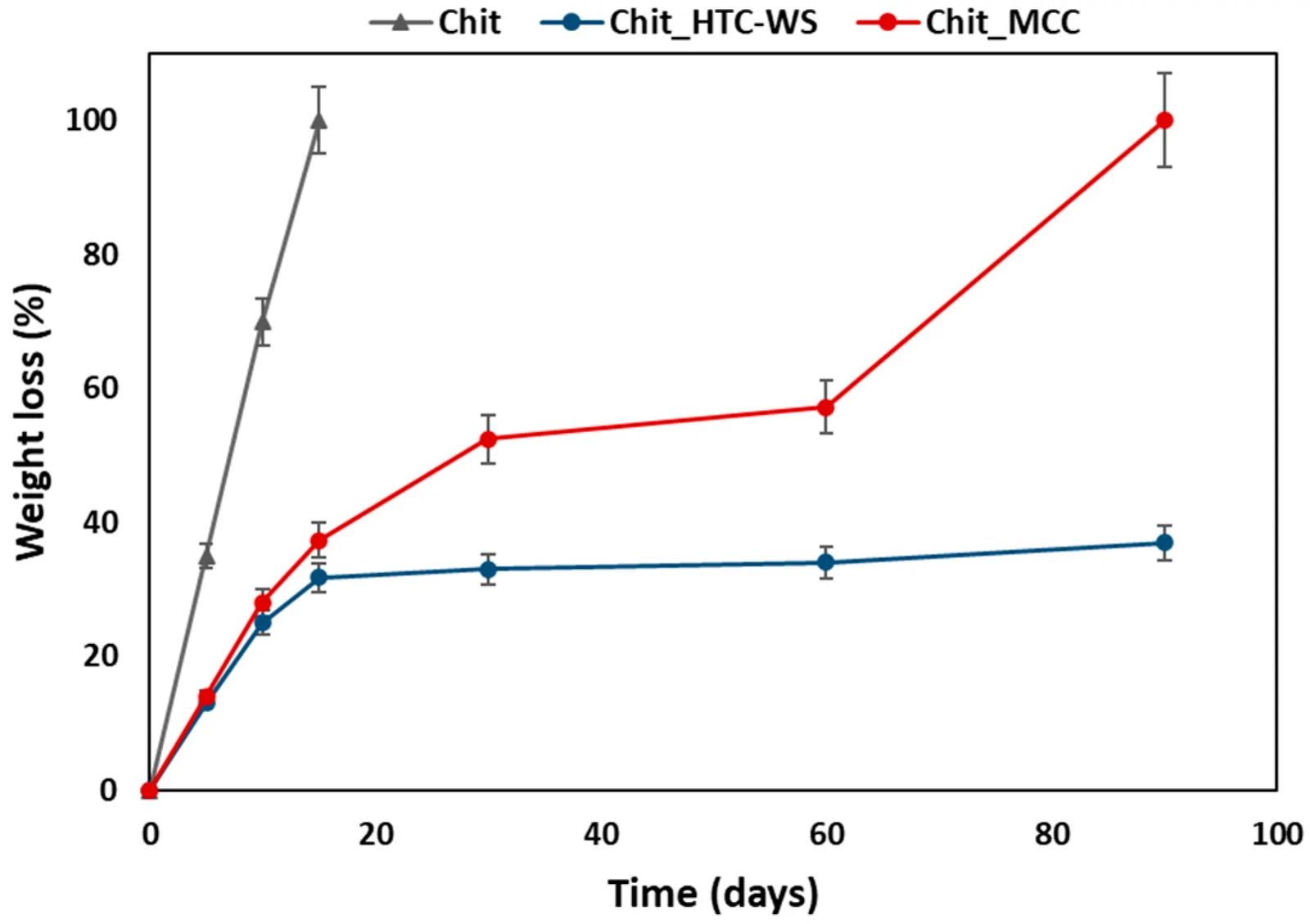
Weight loss of Chit (grey line), Chit_HTC-WS (blue line), and Chit_MCC (red line) films in soil over time.

Enzymatic degradation usually occurs by biodeterioration, fragmentation, assimilation, mineralisation, and metabolism [63]. In soil, microorganisms, including enzymes, bacteria, and fungi, act to increase the degradation rate. The films obtained were subject to degradation in soil, and Figure 11 shows images of Chit, Chit_MCC, and Chit_HTC-WS samples before and after 15 and 60 days, and Figure 12 shows the weight loss (%) of chitosan and chitosan-based films over time.

All films were degraded in soil and maintained at room temperature. The results showed that the neat chitosan film degraded completely within just 15 days. The other films were rough and flexible. Similar results were reported by Wronska et al. [64], suggesting that the initial stage of the chitosan degradation involves depolymerization through random cleavage of β-1,4-glycosidic bonds, followed by deacetylation via hydrolysis of N-acetyl linkages.

A slight weight loss was observed for both Chit_HTC-WS and Chit_MCC films after 2 weeks of exposure (Figure 12). Chit-HTC-WS exhibited greater resistance to degradation compared to Chit_MCC films. The results indicate that the addition of fillers into chitosan films can significantly influence the degradation behavior of the investigated materials.

**Figure 13:**
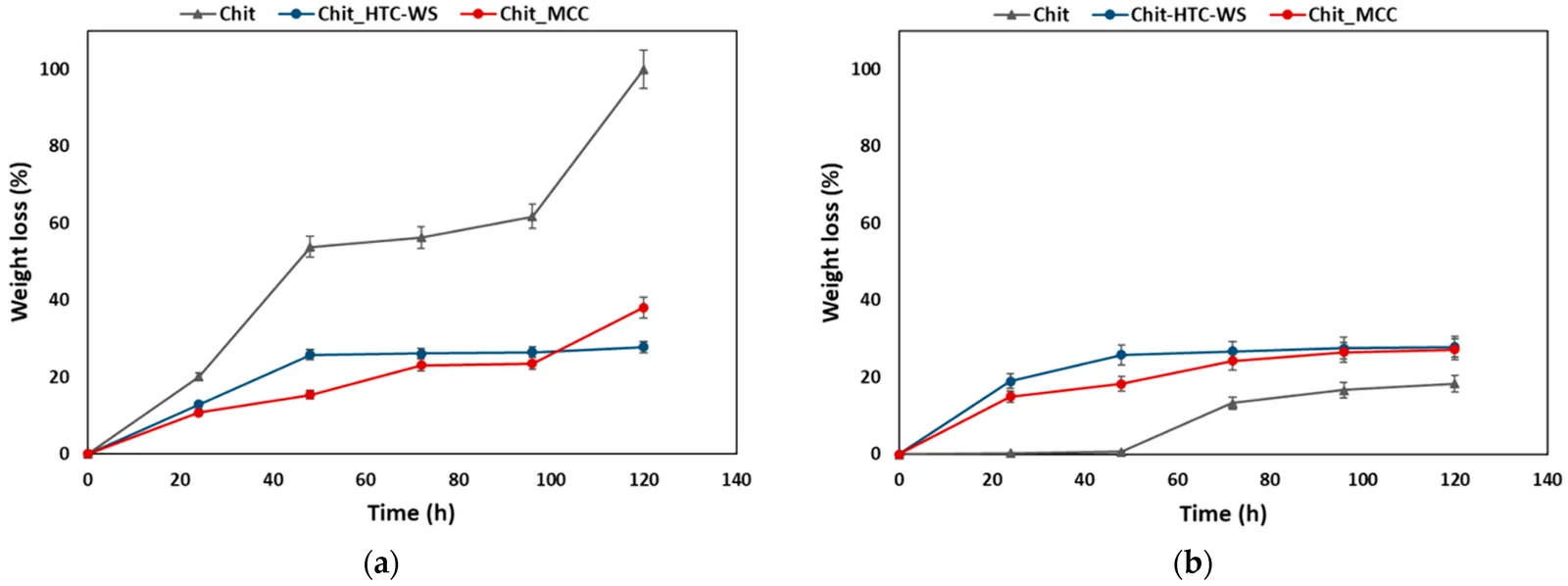
Weight loss of chitosan (grey line), Chit_HTC-WS (blue line), and Chit_MCC films subjected to hydrolytic degradation in (**a**) freshwater and (**b**) simulated seawater over time.

Furthermore, chitosan-based films were subjected to hydrolytic degradation in different environmental conditions, including freshwater and seawater. Figure 13a,b shows the results of weight loss over time.

The films exhibit different behaviour depending on the environment, e.g., freshwater and seawater, and the presence of the fillers. Chitosan is a hydrophilic polymer that absorbs a significant amount of water at room temperature. When neat chitosan film is subjected to hydrolytic degradation in freshwater, a gradual weight loss over time is observed (Figure 13a), with 100% weight loss after 120 h. Julkapli et al. [65] suggested that the degradation of chitosan in distilled water is promoted by the residual –OH and –NH_2_ functional groups present in the film, which promote water uptake through hydrogen-bonding interactions. The Chit_HTC-WS and Chit_MCC films showed substantially lower final mass losses of around 28% and 38%, respectively. This behaviour indicates that the incorporation of both fillers into the biocomposites enhances the stability of chitosan-based films in the aqueous environment. The effect can be related to interactions between the –OH and –COO^−^ groups of the cellulose chains in both HTC-WS and MCC and the –NH_3_^+^ groups of chitosan, which could promote the formation of a less water-permeable structure [65].

In contrast, the neat chitosan film appears to be more resistant to hydrolytic degradation in seawater (Figure 13b), reaching a final weight loss of approximately 18% after 120 h. The lower susceptibility of chitosan to mass loss in the saline medium can be associated with the increased ionic strength of the solution, which screens electrostatic interactions between protonated chitosan chains and modifies their hydration, swelling, and mobility [66]. Furthermore, according to the literature [65], the presence of salts in the simulated seawater resulted in a slightly basic environment, reducing protonation of chitosan amino groups compared with acidic environments, thereby decreasing polymer solubilization.

Interestingly, the influence of salinity was markedly reduced after incorporation of HTC-WS. While neat chitosan film exhibited a pronounced difference between freshwater and simulated seawater, Chit_HTC-WS reached a similar final weight loss in the two media. Hydrogen-bonding interactions between chitosan and HTC-WS can restrict chain mobility and stabilise the polymer network, whereas the hydrochar particles introduce a heterogeneous interfacial structure that provides additional pathways for water penetration. On the contrary, Chit_MCC films exhibited a moderate dependence on the environment, reaching a final weight loss of 38% and 27% in freshwater and seawater, respectively, suggesting that the more heterogeneous nature of HTC-WS plays an additional role in regulating the interaction between the composite and water.

**Figure 14:**
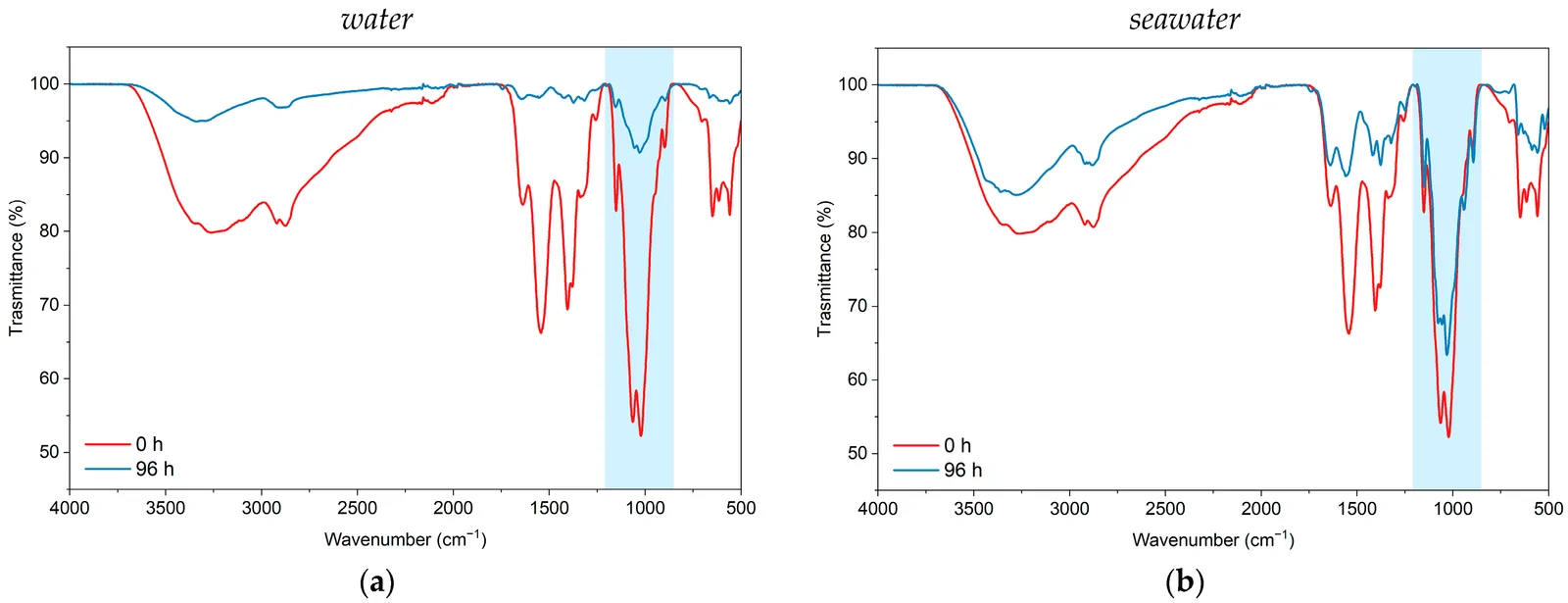
FTIR spectra of chitosan before and after 96 h of immersion in (**a**) water and (**b**) seawater.

**Figure 15:**
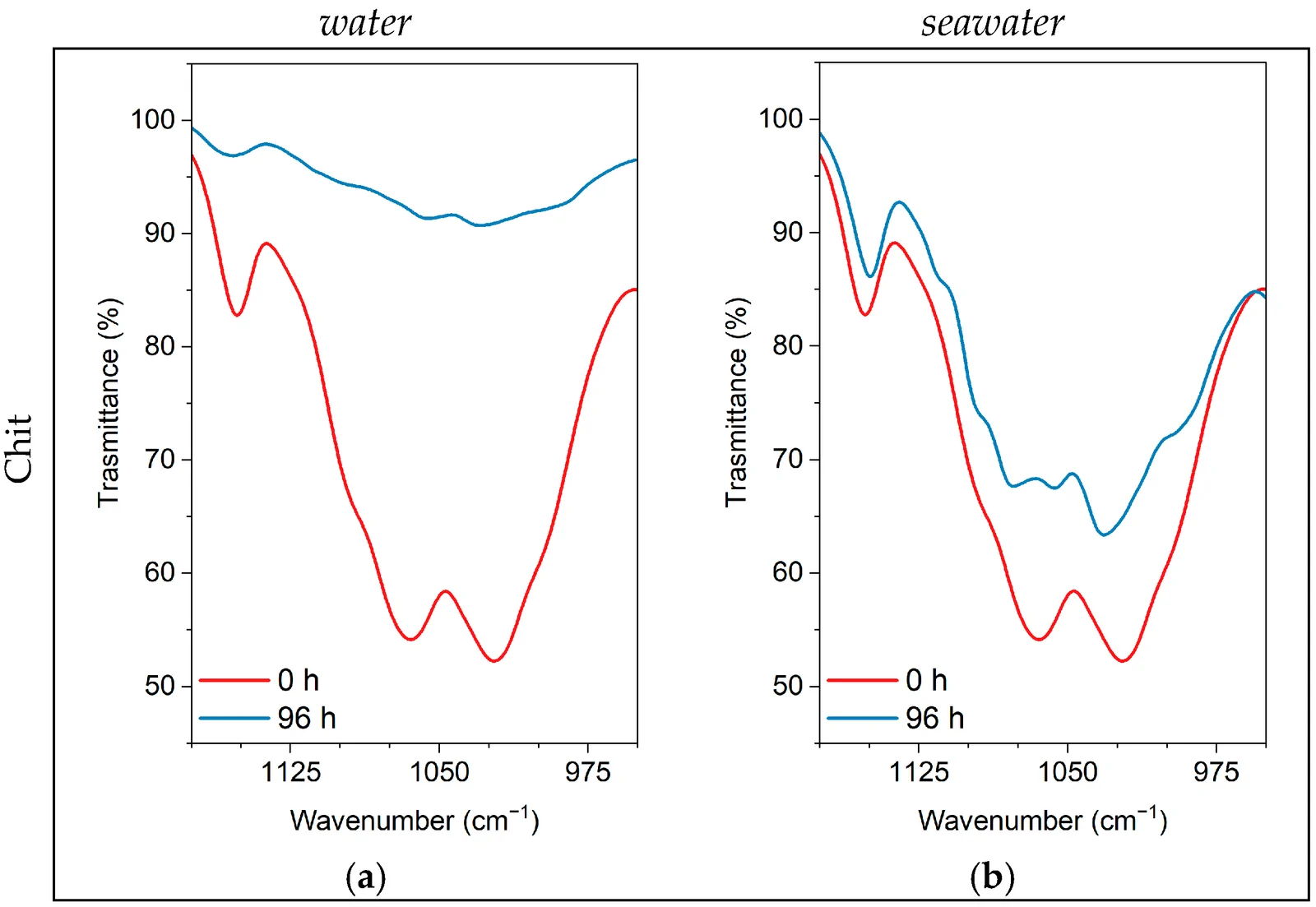

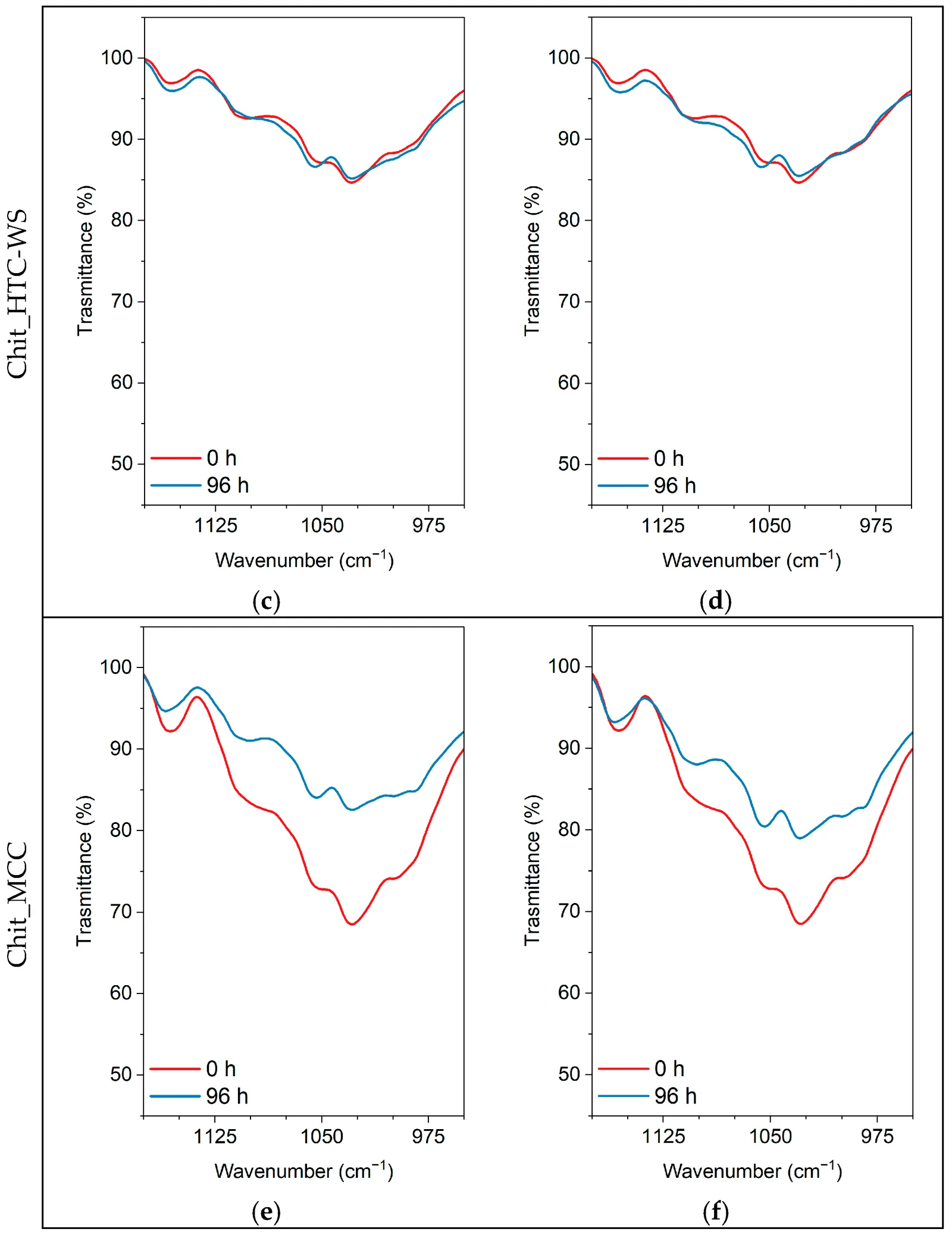
Specific zones in FTIR spectra of (**a**,**b**) Chit, (**c**,**d**) Chit_HTC-WS, (**e**,**f**) Chit_MCC in water and seawater, respectively.

FTIR analysis was performed to investigate possible structural changes occurring during hydrolytic degradation. The spectra were collected before and after 96 h of immersion (note that the chitosan sample at 120 h was completely hydrolysed). Figure 14 shows the FTIR spectra of the chitosan films immersed in distilled water and simulated seawater, while Figure 15 shows the spectra of the chitosan and composite film focusing on the region around 1024 cm^−1^ (blue shaded areas of Figure 14), corresponding to C–O stretching and bending [67].

After 96 h of immersion, the chitosan films (Figure 15a,b) showed a decrease in the intensity of the band in the 1024–1063 cm^−1^ region, which is associated with C–O and C–O–C vibrations of the polysaccharide backbone. This decrease was markedly more pronounced for the chitosan film immersed in distilled water than for the sample exposed to simulated seawater. Such behaviour suggests more extensive structural alterations of the chitosan backbone in freshwater, which may be related to the cleavage of glycosidic C–O–C bonds during hydrolytic degradation. This interpretation is consistent with the higher mass loss observed for the chitosan film in distilled water after 96 h of immersion. In contrast, the Chit_HTC-WS (Figure 15c,d) samples exhibited only minor spectral changes after 96 h in both aqueous environments, with very similar FTIR spectra in distilled water and simulated seawater. These results are consistent with the lower extent of degradation observed for the Chit_HTC-WS films and suggest that the presence of HTC-WS particles contributes to stabilizing the chitosan matrix and reducing its sensitivity to the surrounding salinity.

### 2.4. Environmental Sustainability Considerations of Chitosan-Based Films

The formulation of composite materials based on chitosan and HTC-WS offers significant environmental advantages compared to conventional fossil-derived synthetic materials, in terms of renewable feedstock utilization and potential end-of-life biodegradability.

Moreover, the replacement of commercial MCC with hydrothermally carbonized wood waste (HTC-WS) in chitosan-based biocomposites promotes a more circular use of resources by valorizing agricultural and industrial waste and reducing reliance on chemically intensive cellulose refining processes. While MCC production involves chemical processes such as pulping, bleaching, and acid hydrolysis, HTC-WS is obtained through hydrothermal carbonization, avoiding the use of chemical solvents. The HTC conditions used in this study (210 °C for 1 h) represent a moderate-severity process, limiting excessive energy demand.

From an end-of-life perspective, the replacement of MCC with HTC-WS influences the degradation behavior of the composites. Although Chit_HTC-WS films show a lower degradation rate than Chit_MCC films, the overall degradation time remains within an acceptable range, and the composite film exhibits improved properties compared to the pure chitosan film.

Neat chitosan films and chitosan-based biocomposite films were subjected to accelerated photo-oxidation to simulate degradation phenomena under service conditions.

## 3. Materials and Methods

### 3.1. Materials

Chitosan (Chit), deacetylation degree of 75–85%, low viscosity, and average molecular weight = 120 kg·mol^−1^ was purchased from Sigma Aldrich (St. Louis, MO, USA); hydrochar (HTC-WS) was obtained from pine sawdust waste through hydrothermal carbonization treatment; microcrystalline cellulose (MCC) with an average particle dimensions of 20 μm and bulk density of 0.5 g/mL (25 °C) was purchased from Sigma Aldrich; Glycerol (Gly, 1,2,3-Propanetriol; molecular weight: 92.09 g·mol^−1^), used as plasticizer in this film, and acetic acid (AA, molecular weight: 60.05 g·mol^−1^) were purchased from Sigma Aldrich; Deionized water (DW) was used in the entire study.

### 3.2. Hydrothermal Carbonisation Process

Hydrochar particles (HTC-WS) have been produced through the hydrothermal carbonisation process (HTC), performed in a 500 mL stirred stainless-steel batch reactor (200 rpm). For each experiment, the reactor was loaded with approximately 40 g of sawdust (corresponding to about 37.0 g feedstock on a dry basis d.b.) and about 370.0 g of deionized water (DW), achieving a biomass-to-water ratio of approximately 1:10. After loading, the reactor was sealed, evacuated, and purged three times with high-purity nitrogen to eliminate residual oxygen. The reactor was then heated up to the selected temperature and held under isothermal conditions for the predetermined reaction time.

The process was carried out at a temperature of 210 °C, with a corresponding reaction pressure of about 25 bar and a residence time of 1 h. The reaction conditions, specifically the temperature, were chosen based on the conditions at which the hemicellulose component of the biomass would be completely degraded, while the cellulosic one would still be intact in accordance with the previously reported literature [34].

The solid–liquid HTC reaction mixture (HTC slurry) was separated by vacuum filtration using a pre-weighed cellulose filter. The solid residue on the filter was washed several times with DW to remove the mother liquor and then dried in a ventilated oven at 55 °C until a constant weight was achieved. Dried hydrochar powder was ground and sieved using a 150-mesh sieve, and the resulting material was stored in a sealed container until characterization or use.

### 3.3. Formulation of Biocomposite Films

The biocomposite films were formulated by using the solution casting technique. According to our previous work [53], an aqueous solution of 25 mL containing 2% *v*/*v* acetic acid was prepared. Subsequently, chitosan (0.45 g) was added to the solution and stirred for 20 min at 50 °C until the material was completely dissolved. Hydrochar (HTC-WS, 1.125 g) was then added to the solution and stirred until it was completely dispersed. Glycerol (0.4 g) was subsequently added to enhance the plasticity of the resulting Chit_HTC-WS film. The polymer solution was stirred overnight, then poured into a glass Petri dish and left for 24 h to allow the solvent to evaporate. For comparison, composite films based on chitosan (Chit) and microcrystalline cellulose with glycerol (Chit_MCC) were prepared under similar conditions. The thickness of all the films was around 100 μm.

### 3.4. Characterisations

The biocomposite films were formulated by using the solution casting technique. According to our previous work [53].

#### 3.4.1. Characterisation of Hydrochar

The Malvern Master Sizer 2000 granulometer (Malvern Instruments Ltd., Malvern, UK) was used to analyze the size of the hydrochar particles. The machine was equipped with a Malvern Hydro 2000 MU (Malvern Instruments Ltd., Malvern, UK), useful to disperse 1.0 g of sample into 800 mL of deionized water. All analyses were performed at a stirrer velocity of 2000 rpm, after 5 min of sonication to increase the dispersion. The particle-size distribution curves were obtained from an average of six measurements. Furthermore, d10, d50, and d90, which represent the maximum diameter values of 10%, 50%, and 90% of the particles, respectively, were evaluated.

Proximate analysis was performed according to ASTMD 7582-15 standard [68] using thermogravimetric analysis (Q500 TGA, TA Instruments, New Castle, DE, USA). Around 2–20 mg of sample was placed in a platinum sample pan, held at room temperature under high-purity nitrogen at 60 mL min^−1^ for 15 min. The sample was heated to 105 °C at a rate of 15 °C min^−1^ and held for 20 min to remove moisture. Subsequently, it was heated to 900 °C at the same heating rate and held for 7 min, after which it was cooled to 450 °C at a rate of 15 °C min^−1^. The total mass loss from 105 °C to this point, expressed as a fraction of the total sample mass on a dry basis, was defined as volatile matter (VM) content. The sample was then heated up to 750 °C under an equivalent airflow and held for 15 min. The remaining mass, relative to the dry mass of the sample, was taken as ash content. The fixed carbon (FC) content was calculated on a dry basis by subtracting the VM and ash content from the dry mass.

The hydrochar mass yield (MY_HC_) and gas mass yield (MY_G_) were analysed according to Equations (1) and (2), respectively. The liquid mass yield (MY_L_) was subsequently determined as the complement to 1 of the sum of MY_HC_ and MY_G_ (Equation (3)).

(1) $$\begin{array}{c} {MY}_{HC}=\frac{m_{HC}}{m_{raw}} \end{array}$$

(2) $$\begin{array}{c} {MY}_{G}=\frac{m_{G}}{m_{raw}} \end{array}$$

(3) $$\begin{array}{c} {MY}_{L}=1-\left({MY}_{HC}+{MY}_{G}\right) \end{array}$$

where m_HC_ represents the mass (dry basis) of the solid residue obtained after hydrothermal treatment (i.e., hydrochar, HTC-WS particles), while m_raw_ represents the dry mass of the raw biomass before thermal treatment. Similarly, the gas mass yield, MY_G_, was defined as the mass of gas produced (m_G_) per unit mass of dry raw biomass.

Elemental analysis was performed using a LECO CHN 828 series Elemental Analyzer (LECO Corporation, St. Joseph, MI, USA) to determine the carbon (C), hydrogen (H), and nitrogen (N) content.

Calorimetric analysis to determine the higher heating value (HHV), according to the CEN/TS 14918 standard, was carried out using a LECO AC500 calorimeter (LECO Corporation, St. Joseph, MI, USA), while the proximate analysis, in terms of volatile matter (VM), fixed carbon (FC), and ash content (Ash), was evaluated using a LECO TGA 701 (LECO Corporation, St. Joseph, MI, USA).

The hydrochar energy properties were assessed through the evaluation of the energy yield (EY) and the energy densification ratio (EDR), defined as follows:

(4) $$\begin{array}{c} EY=\frac{{HHV}_{HC}}{{HHV}_{raw}}\cdot {MY}_{HC} \end{array}$$

(5) $$\begin{array}{c} EDR=\frac{{HHV}_{HC}}{{HHV}_{raw}} \end{array}$$

where the HHV_HC_ and HHV_raw_ represent the HHV values of the hydrochar and the raw feedstock, respectively.

The chemical composition of the hydrochar particles was evaluated by using FTIR spectroscopy using a spectrometer equipped with a Micro-ATR objective. Spectra were collected in the attenuated total reflectance (ATR-FTIR) mode over the range of 4000–500 cm^−1^, with a resolution of 4 cm^−1^ and 16 scans, using air as the background.

The microstructure of the obtained particles was examined using a scanning electron microscope (Phenom ProX, Phenom-World, Eindhoven, The Netherlands) operated at an accelerating voltage of 10 kV. The instrument is equipped with a temperature-controlled sample holder maintained at 25 °C. The samples were mounted on an aluminium stub using adhesive carbon tape.

#### 3.4.2. Characterisations of Biocomposite Films

UV-visible spectra were recorded using a UV–visible spectrometer (Specord^®^ 250 Plus, Analytikjena, 24020 Torre Boldine (BG), Italy). Measurements were carried out between 200 and 900 nm with a spectral resolution of 1 nm, averaging eight scans per sample. The transparency (T) values of each film were determined from the absorbance values at 600 nm and calculated according to Equation (6):

(6) $$\begin{array}{c} T=\frac{{Abs}_{600}}{d} \end{array}$$

where Abs600 is the value of absorbance at 600 nm, while d is the film thickness (mm). According to Equation (6), a higher value of T indicates a lower degree of transparency.

Furthermore, the UV-blocking activity was calculated as follows:

U*VX*_*blocking*_ = 100 − *T*_*UV*−*X*_ (7)

where TUV-A and TUV-B represent the average transmittance values in the corresponding spectral regions, specifically UV-A (400–315 nm) and UV-B (315–280 nm), respectively.

Water contact angle measurements were performed using an OCA 20 apparatus (Data Physics Instruments, Filderstadt, Germany), equipped with a CCD camera and a high-performance digitising adapter. Data acquisition and analysis were carried out using SCA 2.0 software (Data Physics Instruments). The films were fixed onto a solid plane support and kept flat during water deposition and acquisition. Measurements were conducted using the sessile drop method with a water droplet volume of 6 µL.

Fourier transform infrared (FTIR) spectra were obtained using a Spectrum One spectrometer (PerkinElmer, Shelton, CT, USA) equipped with a Micro-ATR objective. Measurements were performed in attenuated total reflectance (ATR-FTIR) mode over the spectral range of 4000–500 cm^−1^, with a resolution of 4 cm^−1^ and 16 scans per sample, using air as the background. Spectra were collected for the raw materials: HTC-WS particles, chitosan, and microcrystalline cellulose powders. Additionally, ATR-FTIR analysis was performed on the chitosan-based films to investigate the chemical structure through functional group identification. All measurements were performed in triplicate, and the maximum experimental error, expressed as relative standard deviation, was approximately 5%.

Differential Scanning Calorimetry (Setaram, model DSC131, Mougins, France) was used to investigate the calorimetric properties of the materials. The analysis was conducted using two heating cycles from room temperature to 200 °C at a heating rate of 10 °C min^−1^ and one cooling cycle. For each measurement, approximately 5 mg of the sample were sealed in an aluminium pan.

Mechanical properties were assessed by tensile tests using a Universal Testing Machine (Instron model 3365, Bucks, UK), equipped with a 1 kN load cell, in accordance with the ASTM D882 method [69]. Rectangular samples (10 mm × 90 mm) were cut from films prepared by solvent casting. The tests were carried out at a tensile rate of 1 mm min^−1^, and the elastic modulus (E) was evaluated as the slope of the initial linear region of the stress–strain curves. Average values of elastic modulus, E, elongation at break, EB, and tensile strength, TS, were calculated from seven replicate measurements.

The microstructure of the biocomposite films was examined using a scanning electron microscope (Phenom ProX, Phenom-World, Eindhoven, The Netherlands) operated at an accelerating voltage of 10 kV. The instrument is equipped with a temperature-controlled sample holder maintained at 25 °C. The samples were fractured in liquid nitrogen prior to SEM observation.

### 3.5. Accelerated Weathering and Degradation in Soil, Freshwater, and Seawater

The photo-oxidation resistance of the films (approximately 100 µm thick) was evaluated using a Q-UV-Solar Eye weatherometer (from Q-LAB, Westlake, OH, USA) equipped with UVB lamps. Samples were exposed at 55 °C under an irradiance of 0.89 W m^−2^ at a wavelength of 340 nm and monitored at 24 h intervals. To investigate changes in chemical composition and functional groups during the accelerated weathering, ATR-FTIR analysis was performed on both neat chitosan film and composite films (Perkin-Elmer FT-IR/NIR Spectrum 400, Waltham, MA, USA).

The biodegradation ability of the composite materials in different environments was investigated. The samples were subjected to hydrolysis degradation and soil burial test. Film samples were weighed to determine their initial mass prior to exposure to the different degradation environments.

For the soil burial test, the film samples were buried >20 mm below the soil, and the test was conducted at room temperature for 96 days. At predetermined time intervals, the samples were dug out and gently brushed to remove soil residues. The samples were then thoroughly rinsed with distilled water, dried in an oven at 50 °C until a constant weight was reached, and weighed to determine the percentage of weight loss of the samples.

For biodegradation in aqueous environments, deionized water was used. Simulated seawater was obtained by using salt adapted to marine aquariums (Prodibio, Technopole de Chateau-Gombert, Marseille, France). Laboratory degradation samples were stored at RT in an open-air environment for 120 h, and for all degradation experiments, samples were taken at various intervals of exposure. Before weighing, samples were briefly rinsed with deionized water and dried in an oven at 50 °C until the weight remained constant. The biodegradability was assessed by monitoring weight loss over time.

The weight loss of the biocomposite materials was evaluated by measuring the dry mass of individual samples before and after the degradation, according to the following Equation (8):

(8) $$\begin{array}{c} Weight\;loss\;\left(\%\right)=\frac{m_{i}-m_{r}}{m_{i}}\times 100 \end{array}$$

where m_i_ is the initial dry mass, and m_r_ is the dry mass of the remaining sample that has not decomposed.

## 4. Conclusions

In line with sustainable principles, pine sawdust waste was subjected to hydrothermal carbonisation at 210 °C to produce carbon-rich particles while preserving their cellulose and lignin content. Biocomposite films based solely on naturally occurring constituents, such as chitosan (Chit), wood waste-derived particles (HTC-WS), and microcrystalline cellulose (MCC), were then successfully formulated using the solvent casting technique. The properties and performance of the chitosan-based biocomposite films (e.g., Chit-HTC-WS and Chit-MCC) were compared with those of the neat chitosan film (Chit).

The incorporation of MCC and HTC-WS modified the physicochemical, mechanical, and degradation behaviour of the chitosan films. FTIR spectral changes were consistent with modifications of the hydrogen-bonding environment, while SEM observations suggested favorable filler–matrix contact, particularly for the HTC-WS-containing films. The addition of HTC-WS was also associated with an increase in tensile strength and elongation at break compared with neat chitosan under the investigated formulation conditions.

The Chit_HTC-WS and Chit_MCC films demonstrate UVB protection, extending to UVB blocking, which may be implicated in the enhanced films’ photo-oxidation resistance. Both HTC-WS and MCC enhance the system’s ductility, as demonstrated by a slight increment in tensile strength and elongation at break values. It is evident that both Chit_HTC-WS and Chit_MCC films demonstrate a substantial decrease in the rate of hydrolytic degradation in both environments when compared with the unmodified Chit film. The neat Chit film displays clear evidence of hydrolytic degradation, a process that is significantly influenced by the surrounding environment; following exposure to freshwater, the film undergoes rapid disintegration within 120 h, whereas in simulated seawater, it exhibits complete insolubility. It is noteworthy that neat Chit film is known to disappear during the process of soil degradation after approximately 15 days, whereas a significantly longer period was necessary for the Chit_HTC-WS and Chit_MCC.

Although both Chit_HTC-WS and Chit_MCC films exhibit similar properties and performance, replacing MCC with HTC-WS offers advantages in terms of the films’ thermal behaviour and photo-oxidation resistance. It is also worth noting that HTC-WS could be used as an alternative to commercial microcrystalline cellulose, produced with strong chemical treatments, when formulating chitosan-based biocomposite films.

Therefore, this work is important because it makes it possible to formulate films based on naturally occurring materials and waste materials, subjected to a sustainable hydrothermal carbonised process, that have good properties and are resistant to oxidation. This approach considers the principles of a more sustainable life and uses natural and waste feedstocks for biocomposite film formulations.

## Figures and Tables

**Table 1:** Sawdust and HTC-WS mass yields (wt%, d.b.), HHV (MJ kg^−1^), energy yields EY (%), EDR (%), and proximate and elemental analyses.

	MY_HC_ [%]	MY_G_ [%]	MY_L_ [%]	HHV [MJ/kg]	VM [%]	FC [%]	Ash [%]	EY [%]	EDR [%]	C [%]	H [%]	N [%]
Sawdust	-	-	-	19.82	80.32	19.43	0.25	100	100	46.3	6.52	0.16
HTC-WS	71.8	2.4	25.8	22.55	74.44	25.46	0.10	81.7	113.7	51.4	6.37	0.91

**Table 2:** Calculated values of transparency, UV-A and UV-B blocking activity, and water contact angles (θi) values of the investigated samples.

Sample	Transparency (A mm^−1^)	UV-A Blocking (%)	UV-B Blocking (%)	WCA (°)
Chit	0.40	16.6	25.1	80.5 ± 2.3
Chit_HTC-WS	22.52	100.0	99.9	88.2 ± 2.6
Chit_MCC	7.73	87.1	89.2	92.0 ± 2.5

**Table 3:** Average values of the main thermal properties of raw materials and composite films. (The data on Chit and Chit_MCC films were published before [53]).

Sample	T_on_ (°C)	T_m_ (°C)	T_off_	ΔH (J g^−1^)
HTC-WS powder	38.2	95.2	131.6	189.88
MCC powder	58.4	105.2	144.6	205.14
Chit	48.72	94.1	118.1	161.24
Chit_HTC-WS	96.2	128.2	160.2	231.37
Chit_MCC	74.4	106.4	133.8	147.21

## Data Availability

The original contributions presented in this study are included in the article. Further inquiries can be directed to the corresponding author(s).
